# Application of musculoskeletal ultrasound in postoperative rehabilitation assessment and monitoring after rotator cuff repair: A systematic review

**DOI:** 10.1371/journal.pone.0357622

**Published:** 2026-09-08

**Authors:** Ying Shi, Yi Bao, Chuanxiong Li

**Affiliations:** Department of Rehabilitation Medicine, The Affiliated Hospital of Yunnan University, Kunming, Yunnan Province, China; Shanghai Jiaotong University: Shanghai Jiao Tong University, CHINA

## Abstract

**Background:**

The development of rehabilitation protocols after rotator cuff repair has long lacked objective benchmarks. Traditional time‑based regimens are limited by considerable inter‑individual variability and an increased risk of re‑tear. Musculoskeletal ultrasound allows dynamic assessment of tendon healing and muscle morphology, yet evidence for directly linking its use to rehabilitation decisions remains scarce.

**Objective:**

To systematically synthesize the evidence on the use of musculoskeletal ultrasound monitoring to inform rehabilitation decision‑making after rotator cuff repair.

**Methods:**

Following the Preferred Reporting Items for Systematic Reviews and Meta‑Analyses (PRISMA) guidelines, we searched PubMed, China National Knowledge Infrastructure (CNKI), and Wanfang Data from January 2020 to April 2026. Original studies were included if they involved patients who had undergone rotator cuff repair, used musculoskeletal ultrasound (including gray‑scale ultrasound, elastography, etc.) to evaluate the rotator cuff tendons or shoulder muscles, and reported at least one parameter related to rehabilitation decision-making or functional outcomes.

**Results:**

Eleven studies were included. Shear wave velocity (SWV), cross‑sectional area (CSA), and echo intensity (EI) were the most frequently reported ultrasound parameters. Available evidence indicated that SWV increased progressively after surgery, with an overall increase of approximately 22% to 25% from one week to 12 months postoperatively. This dynamic trajectory may serve as a reference baseline for judging rehabilitation progress. An abnormally elevated SWV in the early postoperative period was associated with an increased risk of re‑tear, suggesting that a more conservative rehabilitation strategy should be adopted. Tendon stiffness measured at 12 weeks after surgery independently predicted long‑term return to sport. Regarding muscle parameters, changes in CSA and EI were positively correlated with shoulder function scores, and the combination of these two parameters effectively identified patients with rehabilitation bottlenecks**.**

**Conclusion:**

Musculoskeletal ultrasound parameters are associated with the initiation of active movement, adjustment of exercise load, prediction of return‑to‑sport prognosis, and identification of retear risk. Among these, SWV shows particular promise as an objective monitoring parameter for supporting rehabilitation assessment after rotator cuff repair. Future randomized controlled trials are needed to determine whether ultrasound-informed assessment can improve rehabilitation outcomes compared with traditional time-based regimens, and to establish standardized measurement protocols and clinically applicable reference values. Key findings of this review are summarized in S1 File.

## 1 Introduction

Rotator cuff tear is one of the most common musculoskeletal disorders [1]. Arthroscopic rotator cuff repair is currently the mainstream surgical treatment for symptomatic full-thickness tears, as it effectively improves shoulder function and reduces pain [2]. Nevertheless, postoperative healing of the repaired tendon and functional recovery are influenced by multiple factors [3], and the design of the rehabilitation protocol is a key determinant of postoperative outcomes [4].

### 1.1 Rehabilitation challenges after rotator cuff repair: Limitations of traditional time‑driven *protocols*

Rehabilitation after rotator cuff repair must balance two conflicting needs: sufficient immobilization to protect the repair interface and promote tendon-to-bone healing, and early mobilization to prevent joint stiffness, muscle atrophy, and adhesion formation. Postoperative shoulder stiffness is a common complication, closely associated with insufficient rehabilitation training and excessive immobilization [5]. Meanwhile, the reported retear rate after rotator cuff repair ranges from 15% to 21%, and can reach as high as 94% in cases of massive tears [6]. A number of systematic reviews and meta-analyses have shown that although retears have a negative effect on functional recovery, the effect size is modest and does not reach the threshold for clinical significance. This means that some patients may still achieve acceptable functional outcomes despite a retear — a finding that suggests postoperative rehabilitation decisions should not be informed solely by the goal of “absolute prevention of retears.” This observation is supported by the evidence from Holtedahl et al. [7]. In addition, age, tear size, fatty infiltration, muscle atrophy, and the appropriateness of the postoperative rehabilitation protocol have all been identified as significant risk factors for retear [8]. Traditional “one-size-fits-all” protocols that rely on fixed time points neglect the substantial inter-individual variability in tear characteristics, tissue quality, and healing trajectory. There is a clear need to shift toward a precision rehabilitation model based on the patient’s individual tissue healing status.

### 1.2 Technical advantages of musculoskeletal ultrasound: non‑invasive, bedside, dynamic, and quantifiable assessment of tendon healing and muscle morphology

Musculoskeletal ultrasound is a non-invasive, real-time, and reproducible imaging modality that has recently demonstrated distinct advantages in the postoperative assessment of rotator cuff repair [9,10]. Advances in high-frequency transducers have enabled clearer visualization of fine tendon structures, panoramic imaging allows larger-scale structural assessment, and the introduction of elastography makes it possible to quantitatively evaluate the biomechanical properties of tendons and muscles [11]. Compared with magnetic resonance imaging, musculoskeletal ultrasound offers several practical benefits, including bedside applicability, dynamic imaging capability, and repeatable follow-up [12]. It also avoids artifacts caused by metal implants, making it particularly suitable for postoperative rehabilitation monitoring [13]. Musculoskeletal ultrasound can be used not only for the differential diagnosis of rotator cuff tears but also for assessing surgical outcomes and supporting rehabilitation monitoring at different postoperative stages [11]. In recent years, ultrasound elastography has also seen important progress in the preoperative evaluation of rotator cuff tears. Zhang et al. (2024) analyzed 106 patients who underwent arthroscopic rotator cuff repair and found that preoperative shear wave velocity (SWV) and elastic modulus were positively correlated with the Constant score at one year postoperatively. The combination of these two parameters predicted postoperative retear with an area under the curve (AUC) of 0.95 and a sensitivity of 91.70%, indicating that preoperative ultrasound parameters can effectively predict long-term functional outcomes and retear risk [14].

### 1.3 Research gap: existing reviews focus on diagnosis or treatment; none have systematically synthesized the evidence linking ultrasound monitoring with rehabilitation-related decisions

In recent years, the application of musculoskeletal ultrasound in rehabilitation after rotator cuff repair has gradually shifted from “structural assessment” to “functional monitoring.” Existing reviews have systematically summarized the normal sonographic appearances of the postoperative rotator cuff and common complications [15], and others have compared the use of ultrasound versus MRI in the postoperative setting [13]. In addition, clinical studies have confirmed that postoperative strain elastography scores are significantly correlated with muscle strength and function [16], that musculoskeletal ultrasound combined with shear wave elastography can assess tendon healing status after surgery for different tear types [17], and that changes in ultrasound parameters are positively correlated with shoulder function recovery [18]. However, most of these studies remain at the level of monitoring; they have not systematically summarized how ultrasound parameters can be directly translated into rehabilitation decisions (e.g., when to initiate active movement, how to adjust load intensity, or how to predict prognosis). In other words, although a large number of studies have used musculoskeletal ultrasound to monitor postoperative rehabilitation, a systematic review specifically focusing on how musculoskeletal ultrasound monitoring may inform rehabilitation decision-making after rotator cuff repair is still lacking.

### 1.4 Study objective

Accordingly, the aim of this systematic review is to systematically search and synthesize the available literature to answer the following core question: which ultrasound parameters, at what postoperative time points, and in what manner have been used to inform adjustments of rehabilitation protocols after rotator cuff repair? By systematically mapping the evidence linking ultrasound parameters to rehabilitation decisions, this review seeks to provide clinicians with evidence‑based support for ultrasound‑informed precision rehabilitation, to identify key gaps in the current evidence base, and to offer directions for future research.

## 2 Methods

This systematic review was designed and reported in accordance with the Preferred Reporting Items for Systematic Reviews and Meta‑Analyses (PRISMA) 2020 statement [19]. The study protocol was registered with PROSPERO (International Prospective Register of Systematic Reviews). The completed PRISMA 2020 checklist is provided in S1 Checklist.

### 2.1 Registered

The protocol for this systematic review was registered with the PROSPERO International Prospective Register of Systematic Reviews (registration number: CRD420261418165).

### 2.2 Search strategy

Searches were conducted in three electronic databases: PubMed, China National Knowledge Infrastructure (CNKI), and Wanfang Data Knowledge Service Platform. The search period covered January 2020 to April 2026, and the language was restricted to English and Chinese.

For PubMed, the following search string was used: (“rotator cuff repair” AND (ultrasound OR elastography) AND (rehabilitation OR decision)).

For the Chinese databases, the search strings were adapted accordingly: CNKI: SU=(’肩袖’ + ‘冈上肌’) * (’修复’ + ‘修补’ + ‘关节镜’) * (’超声’ + ‘肌骨超声’ + ‘弹性成像’) * (’康复’ + ‘术后’ + ‘功能恢复’ + ‘训练’); Wanfang Data: (主题:(肩袖) OR 主题:(冈上肌)) AND (主题:(修复) OR 主题:(修补) OR 主题:(关节镜)) AND (主题:(超声) OR 主题:(肌骨超声) OR 主题:(弹性成像)) AND (主题:(康复) OR 主题:(功能恢复) OR 主题:(术后) OR 主题:(训练)). The complete electronic search strategies are provided in S2 File.

### 2.3 Inclusion and exclusion criteria

The inclusion and exclusion criteria were defined according to the PICOS framework as follows.

#### 2.3.1 Inclusion criteria.

(1) Population: Patients who underwent arthroscopic or open rotator cuff repair, regardless of tear type (full‑thickness or partial‑thickness) or tear size.
(2) Intervention/Exposure: Assessment using musculoskeletal ultrasound, including but not limited to gray‑scale ultrasound, Doppler ultrasound, shear wave elastography (SWE), and real‑time tissue elastography (RTE).
(3) Outcomes: A clear association between ultrasound parameters and either rehabilitation decision‑making (e.g., timing of initiation or progression of active movement, load intensity adjustment, return‑to‑sport determination, retear risk warning) or functional outcomes (e.g., Constant score, ASES score, range of motion, muscle strength).
(4) Study type: Randomized controlled trials, prospective or retrospective cohort studies, case‑control studies, or case series (with a sample size of ≥10 patients).
(5) Publication period: January 2020 to April 2026.
(6) Language: English or Chinese.

#### 2.3.2 Exclusion criteria.

(1) Patients with rotator cuff tears who did not undergo surgical repair (e.g., studies on conservative treatment).
(2) Patients who underwent concurrent shoulder procedures (e.g., Bankart repair, shoulder arthroplasty, biceps tenodesis).
(3) Studies that used only MRI or CT for assessment without performing ultrasound examination.
(4) Studies that reported ultrasound parameters without any form of association with rehabilitation decisions or functional outcomes.
(5) Studies with ineligible study types: case reports (sample size < 10), reviews, editorials, commentaries, conference abstracts, animal experiments, or in vitro studies.

### 2.4 Literature screening and data extraction

Literature screening was performed following the process recommended by the PRISMA 2020 guideline. Duplicate records were first removed using reference management software (NoteExpress). Two investigators independently screened the titles and abstracts against the inclusion criteria and excluded records that clearly did not meet the criteria. Full texts of the potentially eligible articles were then retrieved and independently assessed by the two investigators to make the final decision on inclusion.

Data extraction was also performed independently by the two investigators using a pre‑designed data extraction form. The extracted information included (1) basic study characteristics (first author, year of publication, study design); (2) participant characteristics (sample size, tear type and size, postoperative follow‑up time points); (3) musculoskeletal ultrasound parameters (ultrasound device, parameter types: cross‑sectional area, echo intensity, shear wave velocity, strain ratio, etc.); (4) rehabilitation decision‑related information; and (5) level of evidence. After extraction, the two investigators cross‑checked the data to ensure accuracy. Disagreements between reviewers were resolved through discussion, with a third reviewer consulted when necessary. All screening decisions were reached by consensus; however, formal inter-rater agreement statistics (e.g., Cohen's kappa) were not calculated. The characteristics of included studies are summarized in Table 1.

**Table 1:** Methodological characteristics of included studies. LOE, level of evidence, graded according to the Oxford Centre for Evidence-Based Medicine (CEBM) levels of evidence.

No.	First author, year	Study design	Sample size	Tear type/size	Follow-up points	Ultrasound technique	Key parameters	Decision type / relevance	LOE
1	Itoigawa et al., 2020	Prospective cohort	60	Supraspinatus tears	Preoperative, 1 week, 1–6 months	B-mode + SWE	SWV (muscle/tendon)	Retear warning	2b
2	He et al., 2021	RCT	89	Small/medium supraspinatus	Preoperative, 4, 8, 12, 24 weeks	MSUS (B-mode)	CSA (muscle)	Movement initiation, retear warning	1b
3	Liu et al., 2022	Case series	45	Full/partial tears	3 to 4 months	B-mode + Color Doppler	CSA, EI	Bottleneck ID	4
4	Kim et al., 2023	Retrospective cohort	42	Rotator cuff tear	Postoperative, > 6 months	B-mode + Strain Elastography	Thickness, fluid, SE score	Load adjustment, bottleneck ID	3b
5	Maloof et al., 2023	Prospective cohort	50	Supraspinatus tears	8 days, 6, 12 weeks, 6, 12 months	SWEUS	SWV (tendon)	Load adjustment, return-to-sport	2b
6	Ye et al., 2024	Retrospective cohort	39	Simple supraspinatus	1, 3, 6 months	B-mode + SWE	Thickness, width, SWV	Movement initiation, load adjustment	4
7	Zhu et al., 2024	Prospective observational	95	Mixed tears	Preoperative, 6 months	MSUS (B-mode)	CSA, EI	Movement, load, bottleneck ID	2b
8	Solari et al., 2024	Prospective cohort	48	Rotator cuff tear	1, 6, 12 weeks, 6, 12 months	SWE	SWV (tendon)	Load adjustment, return-to-sport	2b
9	Wang et al., 2025	Retrospective cohort	82	Supraspinatus tear	Preoperative, 8 days	B-mode + Strain Elastography	Thickness, fluid, SE score	Load adjustment, return-to-sport	4
10	Yan et al., 2025	Prospective cohort	73	Rotator cuff tear	Preoperative, 1 week, 1, 3 months	B-mode + SWE	TH, CSA, SWV	Retear warning, load adjustment	2b
11	Maloof et al., 2025	Prospective cohort	50	Supraspinatus tears	8 days, 6, 12 weeks, 6, 12 months	SWEUS	SWV (tendon stiffness)	Return-to-sport/work	2b

### 2.5 Risk of bias assessment

The methodological quality (risk of bias) of the included studies was independently assessed by two reviewers. The assessment tool was selected according to the study design. For the single randomized controlled trial, the Cochrane Risk of Bias tool version 2 (RoB 2) was used, evaluating bias arising from the randomization process, deviations from intended interventions, missing outcome data, measurement of the outcome, and selective reporting of results. For the nine cohort studies, the Risk of Bias in Non-randomized Studies of Interventions tool version 1 (ROBINS-I) was applied, assessing bias due to confounding, selection of participants, classification of interventions, deviations from intended interventions, missing data, measurement of outcomes, and selective reporting of results. For the one case series, the JBI Critical Appraisal Checklist for Case Series was used. Each study was rated as having either “low risk of bias,” “moderate risk of bias,” “high risk of bias,” or “some concerns.” The two reviewers independently performed the assessment and then cross-checked their results. Disagreements were resolved through discussion, with a third reviewer consulted when necessary. For the single randomized controlled trial included in this review, the Cochrane Risk of Bias tool version 2 (RoB 2) was used; the trial (He et al., 2021) was judged to have low risk of bias across all domains. For the case series included in this review (Liu et al., 2022), the JBI Critical Appraisal Checklist for Case Series was used; the study was judged to have moderate risk of bias.

## 3 Results

### 3.1 Study selection

**Fig 1:**
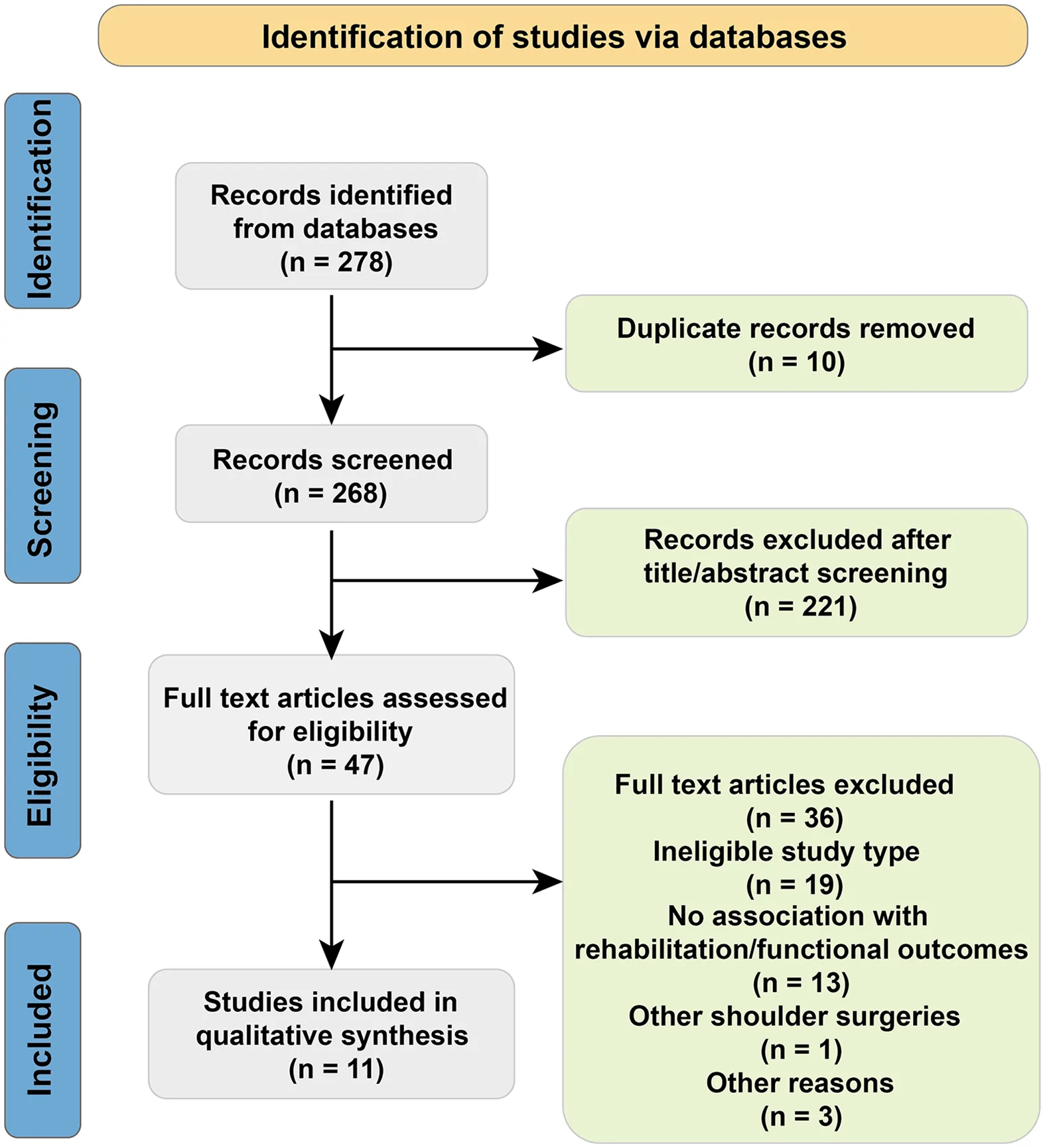
PRISMA 2020 flow diagram of study selection process.

The study selection process was reported following the PRISMA 2020 guidelines. The initial search yielded 278 records: 126 from PubMed, 52 from CNKI, and 100 from Wanfang Data. After removing duplicates using NoteExpress, 268 records remained. Screening by title and abstract excluded 221 records, leaving 47 articles for full‑text assessment. Based on the inclusion and exclusion criteria, 36 articles were excluded after full‑text review. Consequently, 11 studies were included in this systematic review. The screening process and its results are presented in a PRISMA flow diagram Figure 1.

### 3.2 Characteristics of included studies

The 11 included studies were published between January 2020 and April 2026. In terms of study design, there were six prospective cohort studies, three retrospective cohort studies, one case series, and one randomized controlled trial. Sample sizes ranged from 39 to 95 patients, with a total of 673 participants across the included studies. Postoperative assessment time points ranged from one week to 12 months, with one month, three months, and six months being the most common evaluation points.

In terms of ultrasound parameters, six studies focused on tendon parameters (mainly SWV), four studies examined muscle parameters (CSA and EI), and one study reported both types of parameters. Regarding the association with rehabilitation decisions, three studies explored the relationship between ultrasound parameters and the initiation or progression of active movement, seven investigated retear risk warning, four involved prognostic prediction of return to sport, seven addressed load intensity adjustment, and three covered rehabilitation bottleneck identification.

### 3.3 Summary of ultrasound parameters

The included studies primarily reported two categories of ultrasound parameters: muscle morphology parameters (e.g., cross‑sectional area [CSA] of the supraspinatus muscle, used to assess muscle atrophy) and tendon structural parameters (e.g., tendon thickness [TH], shear wave velocity [SWV], used to assess tendon healing quality). A detailed summary of ultrasound equipment and technical specifications is provided in S1 Table.

#### 3.3.1 Tendon parameters.

Six studies reported shear wave velocity (SWV) of the supraspinatus tendon. In normally healed tendons, SWV was approximately 6.20 to 6.30 m/s at one week postoperatively, increased to 7.50 m/s at six months, and reached 7.70 to 7.80 m/s at 12 months [20], representing an overall increase of approximately 22% to 25% over 12 months [21]. In the retear group, SWV at one month postoperatively was significantly higher than that in the healed group (P < 0.05), indicating that an abnormally elevated SWV in the early postoperative period may serve as a warning sign for retear risk [22]. Although some studies evaluated conventional ultrasound parameters such as tendon thickness, most found that these parameters were only weakly associated with postoperative functional recovery and had limited clinical utility. For example, Kim et al. (2023) reported that postoperative tendon thickness showed no significant correlation with the KSS function score [16]. Similarly, Solari et al. (2024) found that although tendon thickness recovered to some extent after surgery, the magnitude of change was much smaller than that of SWV and its correlation with functional scores was weak [20], suggesting that conventional parameters are of limited value in rehabilitation monitoring.

#### 3.3.2 Muscle parameters.

Four studies reported morphological parameters of the supraspinatus muscle. The CSA on the operated side was significantly smaller than that on the contralateral side at six months postoperatively (*P* < 0.001), but the degree of CSA improvement (ΔCSA = postoperative CSA − preoperative CSA) was positively correlated with the Constant score (r = 0.633, *P* < 0.05; 95% CI not reported). The echo intensity (EI) on the operated side was significantly higher than that on the contralateral side at six months (*P* < 0.05), whereas the degree of EI improvement (ΔEI = preoperative EI − postoperative EI) was also positively correlated with the Constant score (r = 0.693, *P* < 0.05; 95% CI not reported) [18]. This indicates that greater improvements in CSA and EI (i.e., larger changes from pre- to post-surgery) are associated with better functional recovery. Liu et al. (2022) followed 45 patients at an earlier time point (3–4 months postoperatively) and similarly found that the CSA on the operated side differed significantly from that on the contralateral side, and that the CSA ratio was positively correlated with shoulder forward flexion, external rotation, and the Constant score, further supporting the value of CSA as a rehabilitation monitoring parameter at different postoperative stages [23].

### 3.4 Summary of how ultrasound parameters inform rehabilitation decisions

#### 3.4.1 Types of decisions.

Based on the available evidence, ultrasound parameters have been associated with five types of rehabilitation decisions.

(1) Initiation and progression of active movement. Restoration of tendon SWV to near-normal reference values suggests that it is safe to progress activity. The normal healing trajectory of SWV reported by Solari et al. (from 6.20 m/s at one week to 7.50 m/s at six months) can serve as a reference baseline for clinical judgment of rehabilitation progression [20].
(2) Load intensity adjustment. Maloof et al. found that tendon stiffness at 12 weeks postoperatively was moderately positively correlated with the ability to return to work and sport at 12 months postoperatively (r = 0.49, *P* = 0.001; 95% CI not reported). Tendon stiffness, measured as shear wave velocity on elastography, reflects the tissue's resistance to deformation and is distinct from the clinical concept of “joint stiffness.” Higher tendon stiffness at 12 weeks was associated with better long-term functional outcomes [10]. This finding suggests that low stiffness may indicate delayed tendon healing. In such patients, premature application of high-intensity loads may increase the risk of retear or delayed healing; therefore, the rehabilitation protocol should be adjusted by reducing load intensity and adopting a more conservative progression strategy. Conversely, patients who reach the target stiffness level can safely and gradually increase training intensity.
(3) Prognostic prediction of return to sport. Tendon stiffness measured at 12 weeks postoperatively is a key prognostic marker that can help identify at an early stage which patients are likely to achieve favorable long-term rehabilitation outcomes (*P* < 0.001) [10].
(4) Retear risk warning. An abnormally elevated SWV at one month postoperatively suggests that a more conservative rehabilitation strategy is needed. In a prospective cohort study of 60 patients who underwent repair of supraspinatus tendon tears, Itoigawa et al. found that muscle SWE values at one month were significantly higher in the retear group than in the healed group (*P* < 0.05). Moreover, in the healed group, SWE values at one month were lower than preoperative values and lower than those measured at four to six months, demonstrating a pattern of early decline followed by gradual recovery; however, as the 4- to 6-month values were not directly compared to the preoperative baseline in the original study, a true V-shaped trajectory cannot be confirmed. In contrast, no such recovery pattern was observed in the retear group [22]. These results support the use of SWE measurement at one month as a warning tool for retear risk, with important clinical value for identifying high-risk patients and adjusting rehabilitation protocols in time to avoid injury.
(5) Rehabilitation bottleneck identification. Poor recovery of CSA or abnormally elevated EI indicates aggravated muscle atrophy or fatty infiltration, which may require the introduction of adjunctive interventions such as neuromuscular electrical stimulation [18].

#### 3.4.2 Decision thresholds.

**Fig 2:**
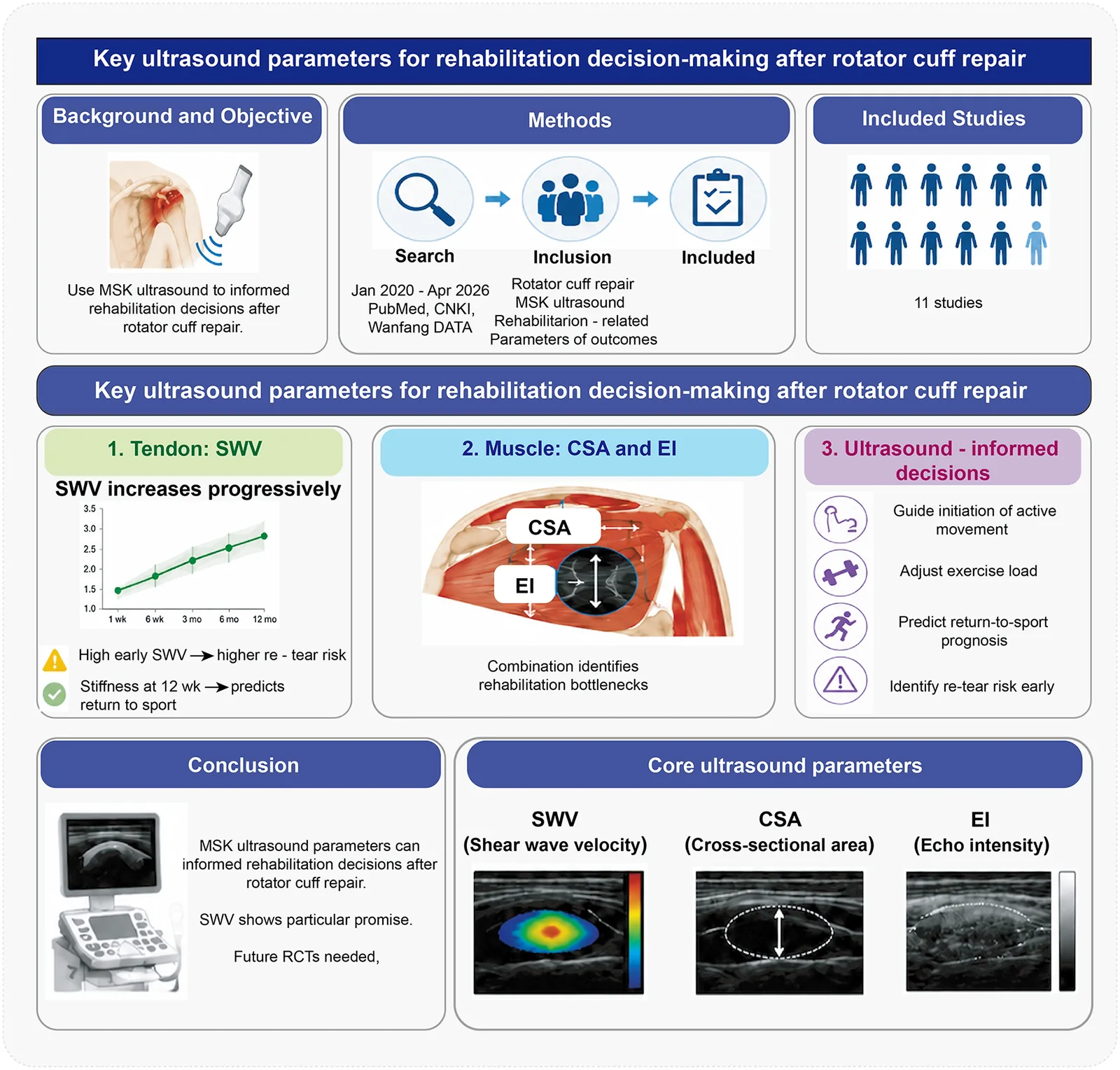
Key ultrasound parameters for rehabilitation decision‑making after rotator cuff repair. MSK, musculoskeletal; RCT, randomized controlled trial; SWV, shear wave velocity; CSA, cross‑sectional area; EI, echo intensity. Created by the authors.

Quantifiable decision threshold information is currently limited. Shimizu et al. established age-stratified reference ranges for the strain ratio. At 0° of abduction, the range was 0.72 to 4.17 for patients younger than 50 years and 0.98 to 4.50 for those 50 years or older. A strain ratio exceeding the upper limit suggests abnormally increased passive stiffness of the tendon and calls for adjustment of the rehabilitation protocol [24]. Solari et al. reported that tendon SWV increased progressively after surgery, from 6.20 m/s at one week to 7.50 m/s at six months (*P* < 0.001). Based on this normal healing trajectory, the authors speculated that a SWV of around 7.50 m/s has been proposed as a potential reference value reflecting postoperative tendon recovery; however, this value has not been validated as a clinical decision threshold [20]. However, this speculation has not yet been validated by prospective studies, and whether 7.50 m/s can serve as a universal reference value remains to be confirmed by further research. Figure 2

### 3.5 Risk of bias assessment results

**Fig 3:**
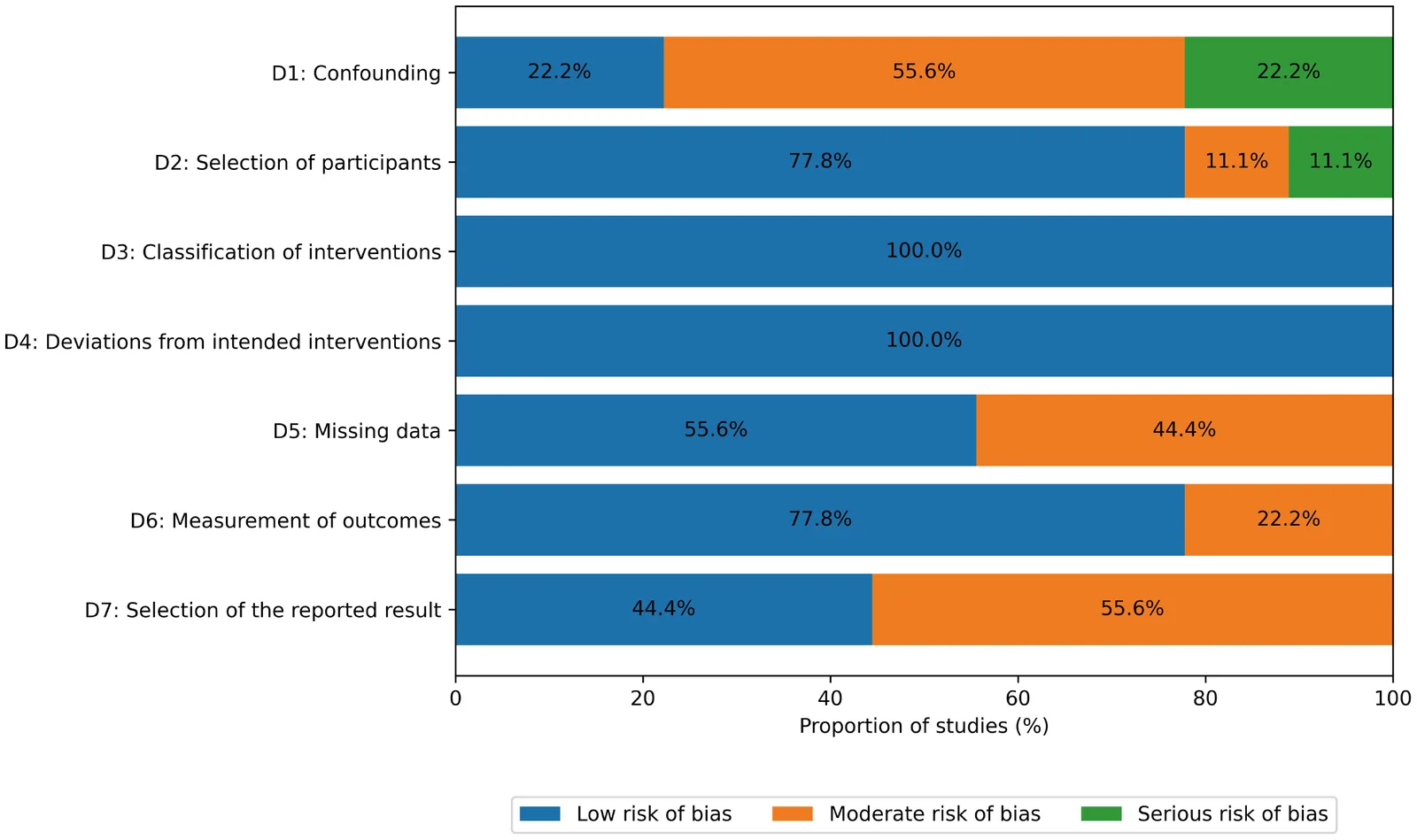
Risk of bias across ROBINS-I domains for the included cohort studies.

**Fig 4:**
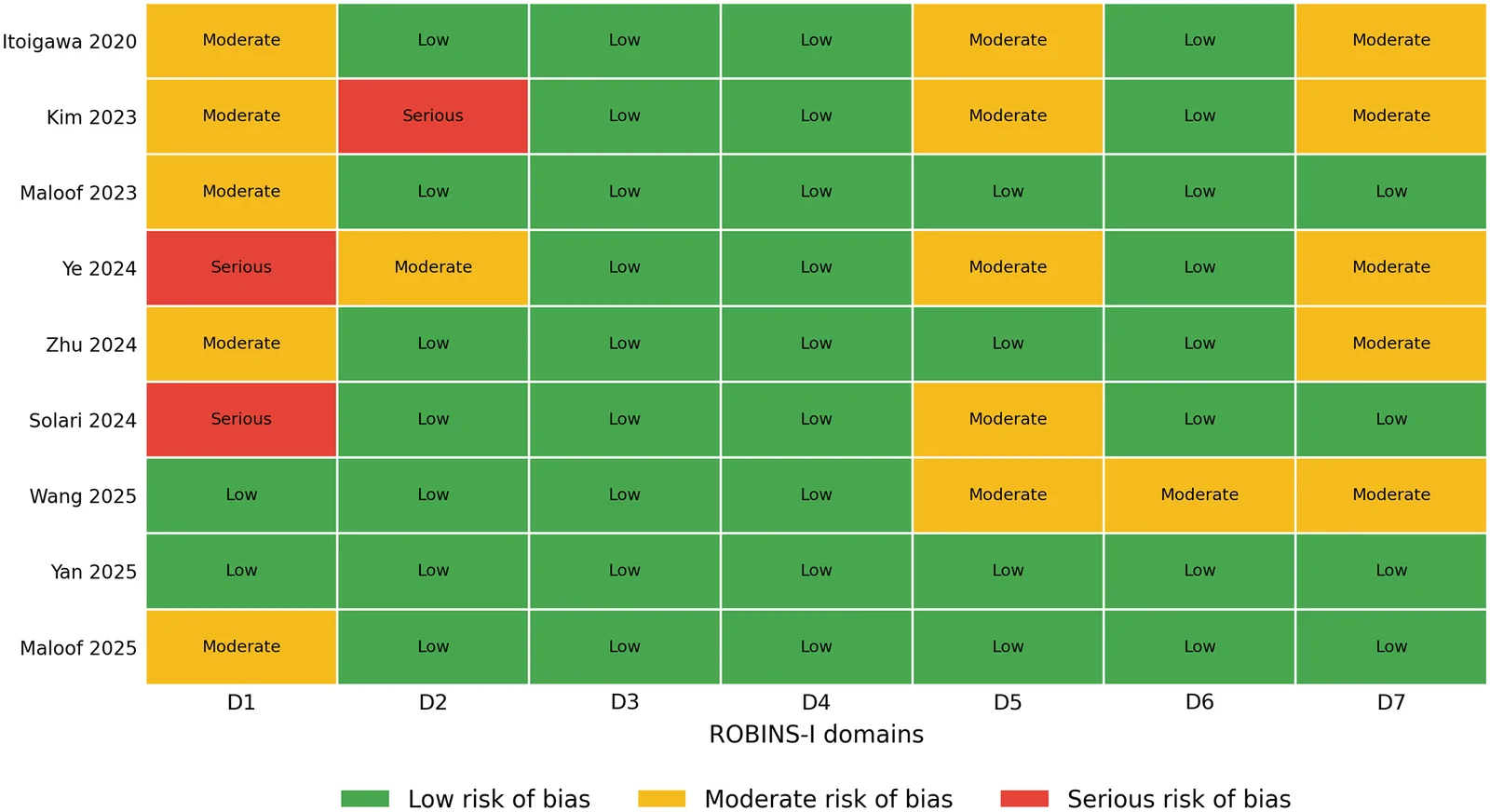
Risk of bias assessment of individual cohort studies using the ROBINS-I tool.

Among the 11 included studies, five were rated as having low risk of bias (He et al., 2021; Maloof et al., 2023; Zhu et al., 2024; Yan et al., 2025; Maloof et al., 2025), and six were rated as having moderate risk of bias (Itoigawa et al., 2020; Liu et al., 2022; Kim et al., 2023; Ye et al., 2024; Solari et al., 2024; Wang et al., 2025). No study was found to have high risk of bias. The main sources of moderate risk of bias included retrospective study design, inadequate adjustment for confounding factors, and failure to report inter-observer agreement for ultrasound measurements. Fig 3, Fig 4.

## 4 Discussion

### 4.1 Summary of main findings

This systematic review is the first to focus on the question of how musculoskeletal ultrasound monitoring can inform rehabilitation decision-making after rotator cuff repair. We systematically synthesized evidence from studies published between January 2020 and April 2026. The main findings can be summarized into three points.

SWV appears to be the most promising quantitative indicator for rehabilitation decision-making. Multiple studies included in this review consistently showed that changes in SWV correlate well with pain relief and functional recovery. A 2024 Chinese study further reported that the AUC of postoperative SWV for distinguishing rehabilitation outcomes using a Constant-Murley score of 70 as the cutoff was 0.781 (95% CI not reported), whereas the AUCs of tendon thickness and width were only 0.531 and 0.466, respectively [25].

One month and 12 weeks postoperatively are two key decision windows, corresponding respectively to retear risk warning and prognostic prediction of return to sport, and can also serve as a basis for adjusting load intensity. A systematic review by Seth et al. (16 studies, 520 patients) reported that SWV at one month postoperatively was significantly higher in the retear group than in the healed group (*P* < 0.05), suggesting that SWE measurement at one month may have potential as a warning indicator for retear risk, although predictive modeling was not performed in the primary studies [26]. Ishikawa et al. (2021) provided parallel evidence from the perspective of muscle activity. Using real-time tissue elastography to follow 20 patients after rotator cuff repair, they found that supraspinatus muscle activity increased markedly between six weeks and three months postoperatively, and by three months it had recovered to a level not significantly different from that of healthy controls. This finding independently validates, from the dimension of muscle activity recovery, the reliability of around 12 weeks (i.e., three months) as a key turning point for functional recovery [27], which is consistent with the study by Maloof et al.

Muscle morphology parameters (CSA and EI) can serve as quantitative indicators of muscle atrophy and fatty infiltration and can be used to identify patients with a “rehabilitation bottleneck.” Zhu et al. (2024) found that improvements in CSA and EI at six months postoperatively were significantly positively correlated with the Constant score (r = 0.633 and 0.693, respectively), and the combination of the two parameters predicted functional recovery with an AUC of 0.922 (95% CI: 0.848 to 0.967) [18]; however, Umehara et al. (2023) showed that even in the absence of obvious degenerative changes in muscle morphology and composition, the active and passive shear moduli of the supraspinatus muscle remained impaired, suggesting that mechanical properties may be more sensitive rehabilitation monitoring indicators than morphological parameters [28].

It should be noted, however, that the lack of correlation between tendon thickness and functional scores reported by Kim et al. (n = 42) should be interpreted with caution. With a sample size of 42, this study had limited statistical power to detect correlations below approximately r = 0.42 (assuming 80% power at α = 0.05, two-sided). Thus, the null finding cannot be taken as evidence of no association, and larger studies are needed to clarify the clinical relevance of morphological parameters.

### 4.2 Comparison with existing reviews

This review differs from the existing literature in three aspects. First, previous reviews have largely focused on the technical application of ultrasound elastography—for example, a 2023 review summarized the potential of SWE in the diagnosis and monitoring of rotator cuff tears [26], and a 2024 systematic review summarized the role of ultrasound elastography in assessing postoperative retear risk [29]. However, none of these reviews took the quantitative link between ultrasound parameters and rehabilitation decision-making as their core research question. Although Sciarretta et al. (2023) emphasized that “rehabilitation protocols should always be individualized,” they did not address how ultrasound imaging could be used to achieve such individualization [30].

Second, this review is the first to systematically map the complete evidence chain from “ultrasound parameter acquisition” to “rehabilitation decision output,” covering both the types of decisions (initiation and progression of active movement, load intensity adjustment, prognostic prediction of return to sport, retear risk warning and rehabilitation downstaging, and rehabilitation bottleneck identification) and decision thresholds, thereby filling the decision-oriented gap in existing reviews. Men et al. (2025) explicitly stated that “musculoskeletal ultrasound can not only be used for the differential diagnosis of rotator cuff tears but also for evaluating surgical outcomes and guiding rehabilitation at different postoperative stages” [11], which is highly consistent with the perspective of the present review. However, that article is a narrative review rather than a systematic review and did not systematically search for or synthesize evidence specifically related to decision-making.

Third, a 2024 Chinese review on “Advances in rehabilitation after rotator cuff surgery” also pointed out that “rehabilitation protocols should be individualized according to the patient's injury and surgical conditions” [31]. However, that review did not address the specific application path of using musculoskeletal ultrasound as a tool for individualization. By systematically synthesizing decision-related examples from primary studies, the present review provides a preliminary evidence framework for translating the concept of “individualized rehabilitation” into the practice of “image-informed precision rehabilitation.”

### 4.3 Clinical implications: feasibility of constructing an “ultrasound‑informed rehabilitation framework”

The value of SWE in informing postoperative rehabilitation advancement has been further substantiated by recent evidence. A systematic review by Lin et al. synthesized data from 11 studies and confirmed that postoperative changes in muscle stiffness, particularly during the 1- to 6-month window, are associated with retear risk and functional recovery, highlighting the potential of sonoelastography to support postoperative monitoring strategies [29]. Complementing this, a prospective cohort study by Yan et al. involving 73 patients demonstrated that SWV at 3 months postoperatively correlated positively with Constant-Murley scores (r = 0.618 to 0.643, *P* < 0.001), providing direct evidence that quantitative ultrasound parameters can serve as an objective imaging basis for tailoring rehabilitation exercise protocols [17]. Together, these findings provide preliminary support for the concept that ultrasound monitoring may provide objective information to support individualized rehabilitation decision-making.

Specifically, the findings of this review support the construction of a precision rehabilitation pathway at three levels. First, at the evidence level. In addition to the dynamic SWV trajectory reported by Solari et al. [20], this judgment has been directly validated clinically. A follow-up study using high-frequency ultrasound and elastography in patients after supraspinatus tendon repair confirmed that SWV has reference value for evaluating pain relief (VAS) and determining rehabilitation outcomes, and may provide clinicians and rehabilitation physicians with additional objective information when developing individualized rehabilitation plans [32].

Second, at the technical level. Musculoskeletal ultrasound is non-invasive, real-time, and reproducible, making it well suited for bedside rehabilitation decision-making. Men et al. (2025) clearly stated that “ultrasound can monitor the repair process of injured tissues and rehabilitation progress in real time during rehabilitation, and by comparing serial images, an individualized rehabilitation protocol can be developed for each patient” [11]. The utility of elastography for assessing tissue biomechanical properties has also been demonstrated in other musculoskeletal conditions, such as plantar fascia assessment in diabetic patients [33].

Third, at the practical level. One study has reported the effect of isokinetic training under musculoskeletal ultrasound monitoring in patients after arthroscopic rotator cuff repair [34], suggesting that ultrasound monitoring can be embedded into routine rehabilitation workflows as an adjunctive tool.

That said, it should be noted that the construction of a precision rehabilitation pathway is still in its early stages. A preliminary attempt has been made to integrate ultrasound monitoring into structured rehabilitation workflows, as illustrated by a study on isokinetic training following arthroscopic repair [34]. Nevertheless, most current studies still follow an “observation-correlation” model (i.e., finding statistical associations between ultrasound parameters and functional outcomes) rather than a “measurement-decision” model (i.e., establishing a direct mapping from ultrasound parameters to rehabilitation instructions). In other words, most research to date has remained at the level of “being able to monitor,” and there is still a considerable methodological gap before ultrasound parameters can be prospectively validated as decision-support tools.

The integration of ultrasound monitoring into rehabilitation decision-making aligns with broader interdisciplinary approaches in sport and health science, which emphasize the synthesis of physiology, biomechanics, and data analytics to develop personalized rehabilitation strategies [35].

### 4.4 Research gaps and future directions

#### 4.4.1 The monitoring value of ultrasound parameters has not been fully exploited in existing *RCTs.*

Saltzman et al. (2017) umbrella review compared the effects of early versus delayed mobilization rehabilitation protocols on pain relief and functional improvement, but it did not involve the use of ultrasound monitoring [36]. He et al. (2021) used CSA as the main outcome measure but did not establish a direct link between specific CSA values and decision boundaries for rehabilitation (e.g., how much CSA recovery is needed to safely progress) [37]. This problem is not an isolated case; it reflects a common shortcoming in the current field. Even in RCT designs, ultrasound parameters are still mainly used as end-point evaluation tools (to determine “which protocol is better”) rather than as real-time decision-guiding tools (to determine “when and how to adjust the protocol”). Future RCTs should embed continuous ultrasound monitoring into the staged decision-making logic of rehabilitation protocols and establish a direct correspondence between ultrasound parameters and rehabilitation instructions.

#### 4.4.2 Integration of patient risk factor stratification with ultrasound‑based decision‑making remains a gap.

In a recent review, Parvizi et al. (2025) reported that the retear rate after rotator cuff repair ranges from 15% to 21%, with risk factors including advanced age, large tear size, poor tissue quality, high activity level, and comorbidities such as diabetes and hyperlipidemia [8]. In 2024, Chen et al., in a prospective study of 89 patients, showed that the preoperative supraspinatus tendon SWV ratio (SWV-RT) was significantly negatively correlated with the arthroscopic remnant tendon quality score (r = −0.722 to −0.884, *P* < 0.001); that is, a lower preoperative SWV indicated poorer intraoperative remnant tendon quality. Based on this finding, the authors suggested that SWE can be used to preoperatively predict remnant tendon quality [38], implying that patients with low preoperative SWV theoretically belong to a “high-risk group” with weaker postoperative healing capacity. However, in current clinical practice, whether patients with different anatomical risk profiles should receive differentiated postoperative rehabilitation progression and intensity (e.g., whether a more conservative SWV decision threshold should be set for patients with poor remnant tendon quality) remains an open question. Although RCTs have explored the effects of different rehabilitation protocols on supraspinatus muscle morphology after rotator cuff repair—for example, He et al. (2021) conducted an RCT (n = 89) comparing informed rehabilitation with conventional rehabilitation on supraspinatus CSA and found that the informed group had superior CSA at all postoperative time points (*P* < 0.05), with no significant difference in retear rates between the two groups (*P* > 0.05) [37]—that study did not answer a more critical question: whether patients with different baseline characteristics (e.g., stratified by baseline CSA, age, or tear size) show heterogeneous responses to the same rehabilitation protocol. In clinical practice, even when a patient has multiple high-risk factors for retear (e.g., a massive tear combined with diabetes), the rehabilitation plan is still based mainly on postoperative SWV values rather than on risk factor-stratified preventive strategies. An integrated closed loop combining risk factor stratification with ultrasound parameter monitoring has not yet been established: whether high-risk patients should be assigned more conservative SWV decision thresholds (e.g., allowing progression only when SWV reaches a certain value at six weeks) remains an untested hypothesis. Future RCTs should incorporate risk factor stratification designs, randomizing patients to either an “integrated SWV plus risk factor” group or a “SWV-only” group, to determine whether integrating risk factors can further improve rehabilitation precision and reduce retear rates in high-risk populations.

#### 4.4.3 Lack of standardized parameters and decision thresholds.

Currently, the ultrasound equipment, transducer frequencies, measurement sites, and region-of-interest (ROI) settings vary considerably across studies, making it difficult to directly compare absolute SWV values between different centers. Moreover, there is a lack of threshold studies oriented toward clinical decision-making—for example, what SWV value would safely allow initiation of active resistance training, or how much CSA reduction would warrant the introduction of electrical stimulation intervention. The age-stratified strain ratio reference ranges established by Shimizu et al. (2023) based on healthy individuals represent an important attempt, but the sample size was small and the clinical decision validity still requires validation in larger samples [24]. Future multicenter collaborative studies are needed to establish standardized ultrasound measurement protocols and a database of clinical outcome-based decision thresholds.

#### 4.4.4 Long‑term follow‑up data are lacking.

Most included studies had follow-up periods of only six to twelve months, and there is a lack of systematic tracking of long-term tendon healing status, functional maintenance, and retear risk beyond one year postoperatively. Of note, RCT-level long-term follow-up studies have already been able to identify structural “residual deficits.” Kjær et al. (2024) performed ultrasound follow-up at one year in 79 patients from the CUT-N-MOVE trial and found that even when rehabilitation was initiated as early as the second postoperative week (compared with the traditional sixth week), the repaired supraspinatus tendon remained significantly thinner than the contralateral side (*P* < 0.001), and muscle thickness was also significantly reduced in the retear subgroup [39]. This high-level evidence indicates that differences in current rehabilitation protocols (early vs. late mobilization) are insufficient to reverse long-term structural deficits, suggesting that the bottleneck for long-term recovery may lie beyond the scope of existing study designs. Future prospective cohort studies of at least two to five years are needed to determine whether the structural deficits observed at one year persist into the second to fifth years and whether they affect the success rate of return to sport.

#### 4.4.5 The biomechanical basis of ultrasound parameters requires further investigation*.*

Among the studies included in this review, the causal mechanisms linking biomechanical parameters of tendons and muscles to clinical functional outcomes remain unclear. Umehara et al. (2023) showed that even in the absence of obvious degenerative changes in muscle morphology and composition, the active and passive shear moduli of the supraspinatus muscle remained impaired, suggesting that mechanical properties may be more sensitive rehabilitation monitoring indicators than morphological parameters [28]. This suggests that future research should shift from merely morphological assessment to a multidimensional monitoring framework that integrates mechanical properties, morphology, and function.

#### 4.4.6 Emerging technologies and decision-support tools*.*

Beyond parameter standardization, emerging technologies such as artificial intelligence (AI) and large language models have shown promise in clinical decision support. Recent studies have explored the application of AI-based tools in delivering guideline-compliant recommendations for musculoskeletal pain management [40] and in comparative evaluations of AI models for clinical scenarios [41]. These approaches may offer new opportunities for integrating serial ultrasound data into rehabilitation decision-making, though their validation in the postoperative rotator cuff population remains an area for future investigation.

### 4.5 *Limitations of this review*

This review has several limitations. First, the included studies were mainly observational, with a lack of large-scale randomized controlled trials, resulting in an overall low level of evidence. Moreover, some of the included studies were case series, which cannot provide dynamic change data, potentially compromising the completeness of the evidence. Second, there was considerable heterogeneity across studies in ultrasound parameter definitions, measurement methods, and follow-up time points, which precluded a meta-analysis and limited the synthesis to a narrative summary. Of note, in the measurement of muscle morphology parameters, the choice of region of interest (ROI) is an important source of heterogeneity. Yuri et al. (2021) specifically addressed this issue by comparing the sensitivity of three different measurement ranges (anterior region, anterior-middle posterior region, and whole cross-sectional area) of the supraspinatus muscle using real-time tissue elastography. They found that the whole cross-sectional area had the highest sensitivity (81.5%) and the best intraclass correlation coefficient (ICC, 0.92 to 0.99) [42]. This quantitative evidence provides a specific operational pathway to resolve the “non-standardized measurement range” issue identified in this review: future studies reporting muscle morphology parameters such as CSA should preferentially adopt the whole cross-sectional area as the standardized measurement range. In addition, although the screening and data extraction were performed independently by two reviewers, formal inter-rater agreement statistics were not calculated, which may limit the reproducibility assessment of the selection process. Third, in terms of sample size, the included studies had sample sizes ranging from 39 to 95 (total 673 participants), with considerable variation between studies, which may have some impact on the comparability of results. Fourth, gray literature and unpublished studies were not included, so there may be potential publication bias. It is worth noting that during the literature screening process, one relevant master's thesis, titled “Application of musculoskeletal ultrasound after rotator cuff repair,” met the inclusion criteria but was excluded because the publication type was restricted to journal articles [43,44]. That thesis pointed out that the retear rate after rotator cuff repair is high, but clinical diagnosis is difficult; although MRI is the gold standard, it is expensive, and therefore musculoskeletal ultrasound can serve as a low-cost, efficient adjunctive examination method to provide a basis for patients’ next-step treatment. Excluding such gray literature ensures a quality benchmark based on peer-reviewed publications, but it may also lead to the omission of high-quality negative results or exploratory findings. Therefore, caution should be exercised when generalizing the conclusions of this review. Fifth, despite optimization of the search strategy, some relevant studies may still have been missed, particularly those in languages other than English or Chinese. Sixth, the risk of bias assessment revealed that among the 11 included studies, six were rated as having moderate risk of bias and five as having low risk of bias. Although no study was judged to have high risk of bias, the proportion of studies with moderate risk of bias was relatively high. Their limitations mainly involved retrospective design, inadequate control of confounding factors, and failure to report measurement consistency in some studies. These factors may have some impact on the overall strength of the evidence, and therefore the conclusions of this review should be interpreted with caution. Despite these limitations, this review is the first to systematically focus on the integration of evidence on “musculoskeletal ultrasound monitoring to inform rehabilitation decision-making after rotator cuff repair,” and it provides a valuable reference framework for future research directions and clinical practice translation in this field.

## 5 Conclusion

This systematic review is the first to synthesize the evidence on the use of musculoskeletal ultrasound monitoring to inform rehabilitation decision-making after rotator cuff repair. The available evidence indicates that ultrasound parameters such as shear wave velocity (SWV), cross-sectional area (CSA), and echo intensity (EI) are associated with initiation of active movement, load adjustment, prognostic prediction of return to sport, and retear risk warning. Among these, SWV appears to be the most promising quantitative indicator, with one month and 12 weeks postoperatively being the key decision windows.

Clinical implications: The synthesized evidence suggests that SWV, CSA, and EI are promising parameters for monitoring tendon healing and muscle recovery after rotator cuff repair. However, specific numerical thresholds for clinical decision-making have not been validated in the included studies. Their use should be confined to research settings until prospective trials establish their validity. Future studies should focus on standardizing measurement protocols and validating parameter-based decision thresholds.

Research recommendations: Future multicenter randomized controlled trials are needed to verify the superiority of ultrasound-informed rehabilitation over traditional rehabilitation, to establish standardized ultrasound measurement protocols and a database of clinical outcome-based decision thresholds, and to extend follow-up to at least 2 years in order to clarify long-term prognosis.

## Supporting information

S1 Table Detailed methodological characteristics of included studies. (DOCX)

S1 File Highlights. (DOCX)

S2 File Search strategy. (DOCX)

S1 Checklist PRISMA 2020 checklist. (DOCX)
