# Supplementary material for: Application of musculoskeletal ultrasound in postoperative rehabilitation assessment and monitoring after rotator cuff repair: A systematic review
Source: PLoS One. 2026 Sep 8;21(9):e0357622. doi: 10.1371/journal.pone.0357622 (PMC13552740; doi:10.1371/journal.pone.0357622)
Supplement: Supplementary file 1 [file pone.0357622.s001.docx]

| **Study** | **Ultrasound System** | **Transducer Frequency** | **Measurement Technique** | **ROI Definition** | **Reported Reliability** |
| --- | --- | --- | --- | --- | --- |
| Itoigawa et al. 2020 | SuperSonic Imagine Aixplorer (version 9.2) | SL10-2 linear array transducer | B-mode + SWE | Repaired tendon: superior side between acromion and just medial to anteromedial anchor hole; Muscle: AD and PD regions from intramuscular tendon to scapular bone | ICC for Goutallier grading = 0.623 (substantial); measurement reliability previously reported as good |
| He et al. 2021 | Philips EPIQ5; Toshiba Xario660 | 10–18 MHz (linear) | MSUS (B-mode) | Supraspinatus CSA measured at midpoint of line connecting lateral and medial points of scapular spine | Not reported |
| Liu et al. 2022 | Philips Q5 | 4–18 MHz (linear) | B-mode + Color Doppler | Supraspinatus CSA measured at midpoint of line connecting lateral and medial points of scapular spine; EI measured using Philips QLab software | Intra- and inter-observer ICC for CSA and EI reported as good |
| Kim et al. 2023 | GE LOGIQ E9 | 6–15 MHz (linear) | B-mode + Strain Elastography | Repaired SST: ROI covering whole width and depth of tendon on short-axis view at 10 mm from footprint; Thickness measured at 10 mm and 20 mm from biceps tendon | Inter-reader agreement: ICC = 0.880 for mean thickness, ICC = 0.980 for subdeltoid fluid; SE scores: κ = 0.828 (almost perfect) |
| Maloof et al. 2023 | Siemens Acuson S3000 | 9L4 transducer (4–9 MHz) | SWEUS | Three points along tendon-bone interface: lateral (at insertion), mediolateral (3 mm medial), medial (6 mm medial); Deltoid muscle (negative control); Humeral head (positive control) | ICC = 0.96 (intra-observer), ICC = 0.45 (inter-observer) from Hackett et al. 2020 |
| Ye et al. 2024 | Siemens Acuson S3000 | 4–9 MHz (linear) | B-mode + SWE | Thickness: long-axis at 10 mm from footprint; Width: short-axis between biceps tendon and infraspinatus; SWV: 1 cm medial to footprint insertion, 1×1 mm ROI | Not reported |
| Zhu et al. 2024 | Philips EPIQ 7 EXP | 10 MHz (linear) | MSUS (B-mode) | CSA measured at midpoint of line connecting lateral and medial points of scapular spine; EI measured using Philips QLab software | Not reported |
| Solari et al. 2024 | Siemens Acuson S3000 HELIX Evolution | 9L4 MHz transducer | SWE | Three ROIs: lateral (adjacent to footprint), 3 mm medial, 6 mm medial; Deltoid muscle (3 cm lateral to anterior acromial insertion); Humeral head (medial to footprint-cartilage junction) | Reliability from Hackett et al. 2020 (ICC = 0.96 intra-observer, ICC = 0.45 inter-observer) |
| Wang et al. 2025 | Philips EPIQ7 | L12-5 probe (5–12 MHz) | B-mode + Strain Elastography | SST thickness: long-axis at 10 mm from footprint; Subdeltoid fluid: maximum diameter on long-axis; SE score: 4-grade visual scoring system | Not reported |
| Yan et al. 2025 | SuperSonic Imagine Aixplorer | SL10-2 probe | B-mode + SWE | TH and CSA: measured at tear site; SWV: ROI (2.0×2.0 mm) placed at tear site (tendon stump for full-thickness tears, sutured site postoperatively), away from anchors | Not reported |
| Maloof et al. 2025 | Siemens Acuson S3000 | 9L4 transducer (4–9 MHz) | SWEUS | Three points along tendon-bone interface: lateral (at insertion), mediolateral (3 mm medial), medial (6 mm medial); Deltoid muscle (negative control); Humeral head (positive control) | ICC = 0.96 (intra-observer), ICC = 0.45 (inter-observer) from Hackett et al. 2020 |

**Supplementary Table S1.** Detailed methodological characteristics of included studies.
