# Supplementary material for: Application of musculoskeletal ultrasound in postoperative rehabilitation assessment and monitoring after rotator cuff repair: A systematic review
Source: PLoS One. 2026 Sep 8;21(9):e0357622. doi: 10.1371/journal.pone.0357622 (PMC13552740; doi:10.1371/journal.pone.0357622)
Supplement: Supplementary file 2 [file pone.0357622.s002.docx]

**Highlights**

MSUS parameters guide rehab decisions after rotator cuff repair.

SWV is the most promising quantitative indicator for rehab.

One month and 12 weeks are key postoperative decision windows.

Lack of standardized thresholds limits evidence-to-practice translation.

First systematic review on MSUS‑guided rotator cuff rehabilitation.
