# Supplementary material for: Application of musculoskeletal ultrasound in postoperative rehabilitation assessment and monitoring after rotator cuff repair: A systematic review
Source: PLoS One. 2026 Sep 8;21(9):e0357622. doi: 10.1371/journal.pone.0357622 (PMC13552740; doi:10.1371/journal.pone.0357622)
Supplement: Supplementary file 3 [file pone.0357622.s003.docx]

Searches were conducted in three electronic databases: PubMed, China National Knowledge Infrastructure (CNKI), and Wanfang Data Knowledge Service Platform. The search period covered January 2020 to April 2026, and the language was restricted to English and Chinese.

The core English database was PubMed, and the following search string was used:(rotator cuff repair AND (ultrasound OR elastography) AND (rehabilitation OR decision)).For the Chinese databases, the following search strings were applied: China National Knowledge Infrastructure (CNKI）:SU=(‘肩袖’ + ‘冈上肌’) * (‘修复’ + ‘修补’ + ‘关节镜’) * (‘超声’ + ‘肌骨超声’ + ‘弹性成像’) * (‘康复’ + ‘术后’ + ‘功能恢复’ + ‘训练’)；Wanfang Data ：(主题:(肩袖) OR 主题:(冈上肌)) AND (主题:(修复) OR 主题:(修补) OR 主题:(关节镜)) AND (主题:(超声) OR 主题:(肌骨超声) OR 主题:(弹性成像)) AND (主题:(康复) OR 主题:(功能恢复) OR 主题:(术后) OR 主题:(训练)).
