# Supplementary material for: Application of musculoskeletal ultrasound in postoperative rehabilitation assessment and monitoring after rotator cuff repair: A systematic review
Source: PLoS One. 2026 Sep 8;21(9):e0357622. doi: 10.1371/journal.pone.0357622 (PMC13552740; doi:10.1371/journal.pone.0357622)
Supplement: Supplementary file 4 [file pone.0357622.s004.docx]

# PRISMA 2020 Checklist (Complete Revised Version)

Prepared based on the current manuscript version: "Application of Musculoskeletal Ultrasound in Postoperative Rehabilitation Assessment and Monitoring After Rotator Cuff Repair: A Systematic Review".

## Item 1: Title

Requirement: Identify the report as a systematic review.

Status / Notes: Completed. The title includes 'A Systematic Review'.

## Item 2: Abstract

Requirement: Provide a structured abstract according to PRISMA requirements.

Status / Notes: Completed. Abstract contains Background, Objective, Methods, Results, Conclusion, and Keywords.

## Item 3: Rationale

Requirement: Describe the rationale for the review in the context of existing knowledge.

Status / Notes: Completed. Introduction describes rehabilitation challenges, ultrasound advantages, and research gaps.

## Item 4: Objectives

Requirement: Provide an explicit statement of objectives or review questions.

Status / Notes: Completed. Section 1.4 states the review objective and core question.

## Item 5: Eligibility criteria

Requirement: Specify inclusion/exclusion criteria and how studies were grouped.

Status / Notes: Completed. PICOS framework and grouping of tendon/muscle parameters are described.

## Item 6: Information sources

Requirement: Specify databases, registers, websites, organisations, and dates searched.

Status / Notes: Completed. PubMed, CNKI, and Wanfang Data are reported. Add exact final search dates if required.

## Item 7: Search strategy

Requirement: Present full search strategies for all databases and limits.

Status / Notes: Completed. Search strategies for PubMed, CNKI, and Wanfang Data are provided.

## Item 8: Selection process

Requirement: Describe study selection methods and reviewer independence.

Status / Notes: Completed. Two reviewers independently screened studies and resolved disagreements.

## Item 9: Data collection process

Requirement: Describe data extraction methods and reviewer independence.

Status / Notes: Completed. Two reviewers independently extracted and cross-checked data.

## Item 10a: Data items

Requirement: List and define outcomes and variables sought.

Status / Notes: Completed. Study characteristics, participants, ultrasound parameters, rehabilitation-related information, and evidence level are extracted.

## Item 10b: Data items / missing information

Requirement: Describe handling of unclear or missing information.

Status / Notes: Recommended addition: No data imputation was performed; analyses were based on reported information.

## Item 11: Study risk of bias assessment

Requirement: Describe risk of bias tools and assessment process.

Status / Notes: Completed. RoB 2, ROBINS-I, and JBI tools are reported.

## Item 12: Effect measures

Requirement: Specify effect measures used for synthesis.

Status / Notes: Completed. Not applicable because this was a narrative systematic review without meta-analysis.

## Item 13a: Synthesis methods

Requirement: Describe how studies were selected for each synthesis.

Status / Notes: Completed. Studies were grouped according to ultrasound parameters and rehabilitation-related outcomes.

## Item 13b: Data preparation

Requirement: Describe preparation or conversion of data.

Status / Notes: Recommended addition: No data conversion was required because narrative synthesis was performed.

## Item 13c: Visual display

Requirement: Describe methods used to display results.

Status / Notes: Completed. Table 1, supplementary tables, and figures present study characteristics and findings.

## Item 13d: Synthesis methods

Requirement: Describe synthesis methods and rationale.

Status / Notes: Completed. Narrative synthesis was selected due to clinical and methodological heterogeneity.

## Item 13e: Heterogeneity exploration

Requirement: Describe methods exploring heterogeneity.

Status / Notes: Completed. Sources of heterogeneity are discussed in limitations.

## Item 13f: Sensitivity analysis

Requirement: Describe sensitivity analyses.

Status / Notes: Not applicable. No sensitivity analysis was performed.

## Item 14: Reporting bias assessment

Requirement: Describe assessment of reporting bias.

Status / Notes: Formal assessment was not performed because the number of studies and heterogeneity limited such analysis.

## Item 15: Certainty assessment

Requirement: Describe assessment of certainty of evidence.

Status / Notes: No GRADE assessment was performed. This is stated as a limitation.

## Item 16a: Study selection results

Requirement: Report search and selection process.

Status / Notes: Completed. PRISMA flow diagram and study selection process are provided.

## Item 16b: Excluded studies

Requirement: Cite studies excluded after full-text review and reasons.

Status / Notes: Consider adding an excluded-study list if requested by the journal.

## Item 17: Study characteristics

Requirement: Cite included studies and present characteristics.

Status / Notes: Completed. Table 1 summarizes all included studies.

## Item 18: Risk of bias in studies

Requirement: Present risk of bias assessment for each study.

Status / Notes: Completed. Table 2 and Figures 3-4 present risk of bias assessments.

## Item 19: Results of individual studies

Requirement: Present results for each included study.

Status / Notes: Completed. Results are presented by ultrasound parameter categories.

## Item 20a: Results of syntheses

Requirement: Summarize study characteristics and risk of bias.

Status / Notes: Completed. Overall findings and risk of bias are summarized.

## Item 20b: Statistical syntheses

Requirement: Present statistical synthesis results.

Status / Notes: Not applicable. No meta-analysis was performed.

## Item 20c: Heterogeneity investigations

Requirement: Present results of heterogeneity investigations.

Status / Notes: Discussed narratively in limitations and research gaps.

## Item 20d: Sensitivity analyses

Requirement: Present sensitivity analysis results.

Status / Notes: Not applicable. No sensitivity analysis was performed.

## Item 21: Reporting biases

Requirement: Present assessment of reporting biases.

Status / Notes: Formal publication bias assessment was not performed and limitation is acknowledged.

## Item 22: Certainty of evidence

Requirement: Present certainty assessment for outcomes.

Status / Notes: No formal certainty grading was performed.

## Item 23a: Discussion interpretation

Requirement: Interpret findings in context of existing evidence.

Status / Notes: Completed. Discussion compares findings with previous reviews.

## Item 23b: Limitations of evidence

Requirement: Discuss limitations of included evidence.

Status / Notes: Completed. Evidence limitations and heterogeneity are discussed.

## Item 23c: Limitations of review process

Requirement: Discuss limitations of review methods.

Status / Notes: Completed. Search limitations and potential bias are discussed.

## Item 23d: Implications

Requirement: Discuss implications for practice, policy, and future research.

Status / Notes: Completed. Clinical implications and future directions are described.

## Item 24a: Registration and protocol

Requirement: Provide registration information.

Status / Notes: Completed. PROSPERO registration is reported.

## Item 24b: Protocol access

Requirement: Indicate where protocol can be accessed.

Status / Notes: Completed. Protocol information is available through PROSPERO.

## Item 24c: Protocol amendments

Requirement: Describe amendments.

Status / Notes: No amendments were reported.

## Item 25: Support

Requirement: Describe financial/non-financial support and funder role.

Status / Notes: Recommended addition: Funders had no role in study design, data collection, analysis, interpretation, or manuscript preparation.

## Item 26: Competing interests

Requirement: Declare competing interests.

Status / Notes: Recommended addition: The authors declare that they have no competing interests.

## Item 27: Data availability

Requirement: Report availability of data, code, and materials.

Status / Notes: Recommended addition: All data generated or analyzed are included in the article and supplementary materials.
