# Supplementary material for: Application of musculoskeletal ultrasound in postoperative rehabilitation assessment and monitoring after rotator cuff repair: A systematic review
Source: PLoS One. 2026 Sep 8;21(9):e0357622. doi: 10.1371/journal.pone.0357622 (PMC13552740; doi:10.1371/journal.pone.0357622)
Supplement: Supplementary file 5 [file pone.0357622.s005.docx]

***Response to Reviewers***

Manuscript ID: PONE-D-26-27615

Title: Application of Musculoskeletal Ultrasound in Postoperative Rehabilitation Assessment and Monitoring After Rotator Cuff Repair: A Systematic Review

***Dear Dr. Liu and Reviewers,***

We sincerely thank the editor and the reviewers for their insightful comments and constructive suggestions on our manuscript. We have carefully considered all the points raised and have revised the manuscript accordingly. Below, we provide our point-by-point responses to each comment.

All changes in the revised manuscript are highlighted in yellow for easy identification. Page numbers refer to the revised manuscript with tracked changes.

***Responses to Journal Requirements***

**Journal Requirement 1:** Please ensure that your manuscript meets PLOS ONE's style requirements.

Response: We have carefully reviewed the PLOS ONE style templates and have formatted our manuscript accordingly, including file naming conventions.

**Journal Requirement 2:** Please ensure that you have uploaded your PRISMA flowchart as Figure 1.

Response: We have uploaded the PRISMA flowchart as Figure 1, as requested.

**Journal Requirement 3:** Please update your submission to use the PLOS LaTeX template.

Response: We have carefully reviewed the PLOS ONE formatting requirements and revised the manuscript accordingly to ensure compliance with the journal’s formatting guidelines.

**Journal Requirement 4 & 5:** The grant information provided in the 'Funding Information' and 'Financial Disclosure' sections do not match. Please remove any funding-related text from the manuscript.

Response: We have removed all funding-related text from the manuscript and ensured that the grant information in the submission system matches the financial disclosure. The correct grant numbers have been verified.

**Journal Requirement 6:** Please provide a complete Data Availability Statement in the submission form.

Response: We have included a complete Data Availability Statement in the submission form. All data presented in this review are derived from the included studies, which are fully cited in the manuscript and supplementary materials.

**Journal Requirement 7:** PLOS requires an ORCID iD for the corresponding author.

Response: The corresponding author's ORCID iD has been validated in Editorial Manager.

**Journal Requirement 8:** Figure 2 contains copyrighted images. Please provide permission or remove.

**Journal Requirement 9:** Please remove your figures from within your manuscript file, leaving only the individual TIFF/EPS image files.

Response: We apologize for the misunderstanding. Figure 2 is created entirely by the authors using Adobe Photoshop. All graphical elements and layouts were created by the authors using original drawings and publicly available software-generated elements.

**Journal Requirement 10:** Please include a new copy of Table 1; the current table is difficult to read.

Response: We have revised Table 1 and improved its readability. Time points have been standardized (e.g., "8 days" instead of "8d"), and all abbreviations have been clarified in the footnotes.

**Journal Requirement 11:** Please include captions for your Supporting Information files at the end of your manuscript.

Response: We have included captions for Supporting Information at the end of the manuscript.

**Journal Requirement 12:** If the reviewer comments include a recommendation to cite specific previously published works, please review and evaluate these publications.

Response: We have carefully reviewed all reviewer-suggested publications and have cited those that are relevant to our manuscript. These citations have been integrated into the appropriate sections of the text.

We are deeply grateful to **Reviewer #1** for the thorough and insightful evaluation of our work. Your meticulous reading and constructive suggestions have been instrumental in improving the clarity and rigor of this manuscript. We particularly appreciate your detailed guidance on the risk-of-bias assessment, sample size verification, and methodological transparency. We have carefully addressed each of your concerns, and we believe the manuscript has been substantially strengthened as a result. Please find our point-by-point responses below.

**Reviewer #1 Comment 1:** The risk-of-bias visual summary does not match the stated methodology. Section 2.5 states explicitly that ROBINS-I was applied to the nine non-randomized cohort studies, yet Figures 3 and 4 present the classic seven-domain Cochrane RoB 1 structure built for randomized trials... The figures need to be regenerated with the correct ROBINS-I domain headings.

Response: We thank the reviewer for identifying this critical inconsistency. We have now thoroughly revised our risk-of-bias assessment figures.

Action Taken: We have regenerated Figures 3 and 4 using the ROBINS-I tool for the nine cohort studies. The figures now display the seven ROBINS-I domains: (1) confounding, (2) selection of participants, (3) classification of interventions, (4) deviations from intended interventions, (5) missing data, (6) measurement of outcomes, and (7) selection of the reported result. The figure titles have also been updated to indicate that the assessments were performed using ROBINS-I.

Location in Revised Manuscript: Figures 3 and 4; Section 3.5.

**Reviewer #1 Comment 2:** The reported total sample size does not reconcile with Table 1. The eleven individual sample sizes listed in the table sum to 665, not the 673 stated in section 3.2.

Response: We apologize for this arithmetic error. We have rechecked all sample sizes against the original publications.

Action Taken: The correct total is 673 (60+89+45+42+50+39+95+48+82+73+50 = 673). We have confirmed that the text in Section 3.2 matches this calculation.

Location in Revised Manuscript: Section 3.2.

**Reviewer #1 Comment 3:** The sample size for He and colleagues' 2021 randomized trial is given twice with two different values. Table 1 lists this study with a sample size of 89, while section 4.4.2 states explicitly that 'He et al. (2021) conducted an RCT (n = 81).'

Response: We appreciate the reviewer's careful reading. We have verified the sample size from the original publication.

Action Taken: The correct figures are: 89 patients enrolled, with 81 completing follow-up. We have clarified in the text that 89 patients were enrolled, with 81 completing the study. In Section 4.4.2, we now refer to the enrolled sample size (n = 89) for consistency with Table 1.

Location in Revised Manuscript: Section 4.4.2.

**Reviewer #1 Comment 4:** References 7 and 8 are duplicates. Both entries read identically... yet they are cited together in the introduction as if they were two independent supporting sources.

Response: We apologize for this oversight.

Action Taken: We have removed the duplicate reference and replaced it with a new relevant reference (Maloof et al., 2025; Orthop J Sports Med). The Introduction now cites only one source [7] for this reference.

Location in Revised Manuscript: References [7] and [8].

**Reviewer #1 Comment 5:** The citation pairing at references 31 and 32 misattributes authorship. The Discussion presents a claim as belonging to 'Xu et al. (2024)' and supports it with the joint citation [31,32], but reference 32 is a 2022 master's thesis by a different author.

Response: We thank the reviewer for pointing out this error.

Action Taken: We have removed both the conference paper (Xu et al., 2024) and the master's thesis (Hu, 2022) from the references, as these publication types were excluded from our inclusion criteria. We have replaced these citations with more robust evidence from peer-reviewed studies: a systematic review by Lin et al. (2024) and a prospective cohort study by Yan et al. (2025). The corresponding text in Section 4.3 has been revised accordingly.

Location in Revised Manuscript: References [31] and [32]; Section 4.3.

**Reviewer #1 Comment 6:** The sentence... introduces low-intensity pulsed ultrasound, a term that appears nowhere else in the paper, without a citation and without any prior mention of a companion protocol. This sentence either needs a proper citation and integration into the argument, or it should be removed.

Response: We agree with the reviewer's assessment.

Action Taken: We have removed this sentence from Section 4.4.5.

Location in Revised Manuscript: Section 4.4.5.

**Reviewer #1 Comment 7:** No inter-rater agreement statistic, such as Cohen's kappa, is reported for either the title and abstract screening stage, the full-text eligibility stage, or the risk-of-bias judgments themselves.

Response: The reviewer raises an important methodological point. We acknowledge that formal inter-rater agreement statistics were not calculated during our screening process.

Action Taken: We have added a statement in Section 2.4 describing how disagreements were resolved through discussion and consensus, with a third reviewer consulted when necessary. Additionally, we have acknowledged this limitation in Section 4.5. While we acknowledge that a formal Kappa statistic would have strengthened the reproducibility assessment, we believe our consensus-based approach with documented discussion of disagreements represents a transparent and methodologically sound alternative.

Location in Revised Manuscript: Section 2.4; Section 4.5.

**Reviewer #1 Comment 8:** A supplementary table listing, for each of the eleven included studies, the ultrasound system manufacturer, model, country of manufacture, transducer frequency and a validation reference for the technique used would directly address the heterogeneity that section 4.4.3 already acknowledges.

Response: We agree that this information is essential for readers to assess the comparability of measurements across studies.

Action Taken: We have created Supplementary Table S1, which summarizes the ultrasound system manufacturer, model, transducer frequency, measurement technique, ROI definition, and reported reliability for each of the 11 included studies.

Location in Revised Manuscript: Supplementary Table S1; Section 3.3.

**Reviewer #1 Comment 9:** None of the quantitative findings summarized from the eleven studies is accompanied anywhere in the manuscript by a confidence interval.

Response: We have addressed this by reviewing the original publications.

Action Taken: For findings where the original studies reported confidence intervals, we have included them. For studies that did not report CIs, we have explicitly noted this (e.g., "95% CI not reported"). Specifically, we have added the CI for the AUC of 0.922 (95% CI: 0.848 to 0.967) from Zhu et al. (2024), and noted where CIs were not available.

Location in Revised Manuscript: Section 3.3.2; Section 3.4.1; Section 4.1.

**Reviewer #1 Comment 10:** The negative finding reported by Kim and colleagues... cannot be interpreted as evidence of absence: with only 42 participants, that study was underpowered to detect anything below a moderate correlation of roughly 0.42.

Response: We agree with the reviewer's interpretation.

Action Taken: We have added a discussion in Section 4.1 noting that the null finding from Kim et al. (n = 42) should be interpreted with caution, as the study was underpowered to detect correlations below approximately r = 0.42 (assuming 80% power at α = 0.05, two-sided). Thus, the absence of a significant correlation cannot be taken as evidence of no true association.

Location in Revised Manuscript: Section 4.1.

**Reviewer #1 Comment 11:** The header row of Table 1 contains the compressed label 'Tear typesize'... The Limitations section contains a near-duplicate sentence... The parenthetical aside built around a long dash... Decimal and formatting verification...

Response: We have carefully revised all language and punctuation issues identified by the reviewer.

Action Taken:

Table 1 header corrected to "Tear type/size"

Duplicate sentence in Section 4.5 removed

Long dashes revised into separate sentences

Decimal formatting standardized to two decimal places for SWV values (e.g., "6.20 to 6.30 m/s")

Range expressions standardized to use "to" consistently (e.g., "15% to 21%")

Location in Revised Manuscript: Table 1; Section 4.5; Section 3.3.1.

**Reviewer #1 Comment 12:** The introduction... opens with the unreferenced assertion that 'rotator cuff tear is one of the most common musculoskeletal disorders.'... [The authors should cite Oliva et al., I.S.Mu.L.T. Guidelines, Padulo et al., etc.]

Response: We thank the reviewer for these valuable suggestions.

Action Taken: We have incorporated the recommended references:

Oliva et al. (2014) to support the epidemiology statement

I.S.Mu.L.T. Rotator Cuff Tears Guidelines (2016) to support arthroscopic repair as mainstream treatment

Padulo (2025) in Discussion 4.3 for interdisciplinary approaches

Santoboni et al. (2023) in Discussion 4.3 as a methodological parallel

Location in Revised Manuscript: References [41]-[44]; Section 1; Section 4.3.

**Reviewer #1 Comment 13:** The PROSPERO registration number given (CRD420261418165) contains fifteen characters, one more than the fourteen-character format... The authors should re-verify this number.

Response: We have verified the PROSPERO registration number.

Action Taken: The registration number CRD420261418165 is correct and matches our PROSPERO registration record. The 15-character format is valid for PROSPERO IDs.

Location in Revised Manuscript: Section 2.1.

We sincerely thank **Reviewer #2** for the thoughtful and constructive feedback on our manuscript. Your critical assessment of the alignment between our research question and the available evidence was especially valuable. We have carefully reconsidered our language throughout the manuscript, shifting from "guide" to "inform" and "associate with" to more accurately reflect the nature of the evidence. We have also incorporated your suggestions on the AI/large language model perspective and clarified the methodological limitations you raised. We are confident that these revisions have significantly improved the manuscript. Please find our detailed responses below.

**Reviewer #2 Comment 1:** The stated aim is to determine 'which ultrasound parameters ... have been used to guide adjustments of rehabilitation protocols.' Yet the 11 included papers predominantly report statistical associations between MSUS parameters and functional scores... The conclusion that MSUS 'can guide the initiation of active movement...' is therefore not supported by the included evidence.

Response: The reviewer raises a critical point. We acknowledge that the evidence base consists primarily of observational studies reporting associations, not interventional studies where ultrasound findings directly triggered protocol changes.

Action Taken: We have systematically revised the language throughout the manuscript to accurately reflect the nature of the evidence. Specifically, we have replaced terms such as "guide" and "guided" with more appropriate terms such as "associate with," "inform," and "provide a reference for." The title, abstract, main text, and conclusion have all been revised accordingly. We have also emphasized in the Discussion that most current studies follow an "observation-correlation" model rather than a "measurement-decision" model. We have also carefully removed remaining causal wording and emphasized that current evidence supports association and decision support rather than direct ultrasound-triggered rehabilitation protocols.

Location in Revised Manuscript: Abstract; Section 1.4; Section 3.4; Section 4.1; Section 4.3; Section 5.

**Reviewer #2 Comment 2:** Zhao XQ et al. 2025 appears identically as references [7] and [8].

Response: We apologize for this error. As detailed in our response to Reviewer #1 Comment 4, we have removed the duplicate reference and replaced it with a new relevant citation (Maloof et al., 2025).

Location in Revised Manuscript: References [7] and [8].

**Reviewer #2 Comment 3:** The master's thesis (Xie Y, ref [40]) and conference paper (Xu M, ref [31]) are excluded from the synthesis yet are cited in the discussion as supportive evidence.

Response: We agree with the reviewer that this was inconsistent.

Action Taken: As detailed in our response to Reviewer #1 Comment 5, we have removed the conference paper (Xu et al., 2024) and the master's thesis (Hu, 2022) from the references and replaced them with peer-reviewed journal articles (Lin et al., 2024; Yan et al., 2025). The master's thesis (Xie Y, 2021) is still referenced in Section 4.5 only as an example of excluded gray literature, which is consistent with its status.

Location in Revised Manuscript: References [31] and [32]; Section 4.3; Section 4.5.

**Reviewer #2 Comment 4:** In the abbreviation list, KSS is defined as 'Knee Society Score' — an obvious copy-paste mistake for a shoulder study.

Response: We apologize for this oversight.

Action Taken: We have corrected the abbreviation to "Korean Shoulder Score."

Location in Revised Manuscript: Abbreviations section.

**Reviewer #2 Comment 5:** In section 4.4.1, Saltzman et al. is called a '2023 umbrella review', but reference [35] is the 2017 paper.

Response: Thank you for catching this error.

Action Taken: We have corrected the year to 2017.

Location in Revised Manuscript: Section 4.4.1.

**Reviewer #2 Comment 6:** Seth et al. (2023, ref [25]) is said to confirm 1-month SWV as predictor; the original paper reported significance but explicitly stated no predictive modelling was done.

Response: The reviewer is correct. We have revised our description to accurately reflect the original study's findings.

Action Taken: We now state that Seth et al. "reported" the difference rather than "confirmed" it, and we explicitly note that "predictive modeling was not performed in the primary studies."

Location in Revised Manuscript: Section 4.1.

**Reviewer #2 Comment 7:** Maloof et al. 2023 and Maloof et al. 2025 may represent overlapping cohorts but are treated as separate studies.

Response: We acknowledge this concern.

Action Taken: In Supplementary Table 1, we have noted that both studies come from the same institution using the same sonographer and ultrasound system, and that while patient overlap cannot be definitively excluded based on published data, they were treated as separate studies due to their distinct research questions and follow-up analyses.

Location in Revised Manuscript: Supplementary Table 1.

**Reviewer #2 Comment 8:** The closing clinical recommendation states: 'When SWV deviates from the normal healing trajectory (e.g., < 7.0 m/s at 3 months or < 7.5 m/s at 6 months)...' Neither the 7.0/7.5 m/s values nor the 20% CSA cut-off are reported by any included primary study. These are author inventions.

Response: The reviewer is correct. These thresholds were not derived from the included studies.

Action Taken: We have removed all specific numerical thresholds from the Conclusion. The revised text now states that SWV, CSA, and EI are promising parameters, but that specific numerical thresholds have not been validated and should be confined to research settings until prospective trials establish their validity.

Location in Revised Manuscript: Section 5.

**Reviewer #2 Comment 9:** Title page lists 'Ying SHI, Chuanxiong LI, Yi BAO'; main text lists 'Ying Shi, Yi Bao, Chuanxiong Li'. Inconsistent.*

Response: We have verified the author order.

Action Taken: Both the title page and the main text now consistently list the authors as Ying Shi, Yi Bao, and Chuanxiong Li (corresponding author). The same order will be entered in the submission system.

Location in Revised Manuscript: Title page; CRediT statement.

**Reviewer #2 Comment 10:** The LOE column in Table 1 uses 1b/2b/3b/4 without stating the grading system.

Response: We agree that this clarification is needed.

Action Taken: We have added a footnote to Table 1 indicating that LOE is graded according to the Oxford Centre for Evidence-Based Medicine (CEBM) levels of evidence.

Location in Revised Manuscript: Table 1.

**Reviewer #2 Comment 11:** Replace 'guide rehabilitation decision-making' with 'association with rehabilitation decision points' in title, abstract, and headings.

Response: We agree with this suggestion and have implemented it throughout.

Action Taken: We have systematically revised language throughout the manuscript to reflect associations rather than causal guidance. This affects the abstract, title, headings, main text, and conclusion.

Location in Revised Manuscript: Abstract; Section 1.4; Section 3.4; Section 4.1; Section 4.3; Section 5.

**Reviewer #2 Comment 12:** The potential role of AI/Large language model in rehabilitation decision-making should be addressed in the future outlook section... It is suggested that these studies be cited (PMID: 40597769, PMID: 40952435).

Response: We thank the reviewer for this forward-looking suggestion.

Action Taken: We have added a new subsection 4.4.6 (Emerging technologies and decision-support tools) discussing emerging AI and large language model technologies in rehabilitation decision-support. We have cited both recommended references (Deng et al., 2025; Dong et al., 2025).

Location in Revised Manuscript: Section 4.4.6; References [43] and [44].

We greatly appreciate Reviewer #3 for the thoughtful and detailed review of our work. Your careful analysis of the methodological rigor, the logical coherence of our conclusions, and the strength of our clinical recommendations was extremely helpful. We have addressed each of your concerns, particularly regarding the healing trajectory logic, the provenance of reference values, the discussion of cost-effectiveness and safety, and the clarification of correlation versus causation. We have also improved the structure of the Discussion section as you suggested. We believe these revisions have made the manuscript much more robust and transparent. Please find our point-by-point responses below.

**Reviewer #3 Comment 1:** Why only analyze the literature from PubMed, CNKI and Wanfang, which may cause analysis bias? A review that aims to synthesize general evidence should ideally include major international databases such as Web of Science.

Response: The reviewer raises a valid concern about potential selection bias.

Action Taken: We have acknowledged this limitation in Section 4.5, noting that the search was restricted to PubMed, CNKI, and Wanfang Data, and that the exclusion of Web of Science and Scopus may have introduced selection bias. However, we also note that the inclusion of both English and Chinese databases was intended to capture a broad range of studies given the review's focus on both international and Chinese literature.

Location in Revised Manuscript: Section 4.5.

**Reviewer #3 Comment 2:** The conclusion that the healed group showed a 'first decrease, then increase' healing trajectory is logically flawed... To establish a true V-shaped trajectory, it is essential to demonstrate that the 4-6 month SWV has returned to a level equal to or higher than the preoperative baseline.

Response: The reviewer's logical analysis is correct. We have revised our description to accurately reflect what the data actually show.

Action Taken: We now describe this as a "pattern of early decline followed by gradual recovery," and we explicitly note that "as the 4- to 6-month values were not directly compared to preoperative baseline in the included studies, a true V-shaped trajectory cannot be confirmed and requires further investigation."

Location in Revised Manuscript: Section 3.4.1.

**Reviewer #3 Comment 3:** It is unclear whether these two studies are from the same center, using the same equipment and operator... Presenting findings from one or two studies as a 'reference baseline' overstates the evidence.

Response: We agree that we overinterpreted these findings.

Action Taken: We have revised the text to describe these findings as a "preliminary reference" rather than a definitive baseline, and we note that these findings originate from a single center and require validation across different equipment and operator settings.

Location in Revised Manuscript: Section 3.4.1.

**Reviewer #3 Comment 4:** In the Conclusion section, the authors recommend that 'when SWV falls below 7.0 m/s at 3 months or 7.5 m/s at 6 months, the rehabilitation protocol should be adjusted.' ... Placing unvalidated numerical cutoffs in the conclusion risks premature clinical adoption.

Response: The reviewer is entirely correct. These thresholds were not derived from the included studies.

Action Taken: As detailed in our response to Reviewer #2 Comment 8, we have removed these thresholds from the conclusion. The revised conclusion now emphasizes that while SWV, CSA, and EI are promising parameters, specific numerical thresholds have not been validated and should only be used in research settings until prospective trials establish their validity.

Location in Revised Manuscript: Section 5.

**Reviewer #3 Comment 5:** The authors recommend routine ultrasound examinations at 1, 3, and 6 months postoperatively. However, no consideration is given to cost-effectiveness, patient adherence, or the actual rate of rehabilitation protocol change resulting from these examinations.

Response: The reviewer raises important implementation considerations.

Action Taken: We have added a statement in Section 4.3 noting that the proposed monitoring strategy requires consideration of cost-effectiveness, patient adherence, and the frequency with which ultrasound findings actually lead to protocol modifications. These implementation factors were not addressed in the included studies and represent an important area for future health services research alongside clinical validation.

Location in Revised Manuscript: Section 4.3.

**Reviewer #3 Comment 6:** The authors do not address whether ultrasound-guided protocols can reduce retear rates without increasing the risk of joint stiffness or delayed recovery—both of which are critical safety endpoints.

Response: We agree that this safety balance is an important unanswered question.

Action Taken: We have added a paragraph in Section 4.3 acknowledging that a critical unanswered question is whether ultrasound-informed rehabilitation protocols can reduce retear rates without increasing the risk of joint stiffness or delayed recovery. The current evidence base is insufficient to evaluate this safety balance, and future randomized trials should incorporate both retear and range-of-motion endpoints.

Location in Revised Manuscript: Section 4.3.

**Reviewer #3 Comment 7:** The Discussion currently jumps from 'main findings' to 'comparison with existing reviews' to 'clinical pathway feasibility' to 'research gaps' without a clear narrative thread.

Response: We have reviewed the Discussion structure and made improvements.

Action Taken: We have retained the existing structure (4.1 Summary, 4.2 Comparison, 4.3 Clinical implications, 4.4 Research gaps, 4.5 Limitations) but added transitional sentences between sections to improve narrative flow. Specifically, we added a transition from 4.2 to 4.3 and from 4.3 to 4.4.

Location in Revised Manuscript: Section 4.2; Section 4.3.

**Reviewer #3 Comment 8:** The authors state that 'the combination of CSA and EI effectively identified patients with rehabilitation bottlenecks.' However, the cited studies are cross-sectional or short-term observational designs that only demonstrate correlation, not causation.

Response: The reviewer is correct. We have revised the language throughout to reflect associations rather than causal relationships.

Action Taken: In Section 3.3.2, we now use "is associated with" rather than "indicates," and we explicitly note that causal relationships between imaging findings and functional limitations have not been established.

Location in Revised Manuscript: Section 3.3.2.

**Reviewer #3 Comment 9:** References 7 and 8 are identical duplicates. Please remove one and also check other references.

Response: As detailed in our response to Reviewer #1 Comment 4 and Reviewer #2 Comment 2, this has been resolved.

Location in Revised Manuscript: References [7] and [8].

**Reviewer #3 Comment 10:** Although Section 3.2 provides a textual description... there is no comprehensive table summarizing key characteristics such as sample size, patient demographics, tear type/size, surgical approach, follow-up time points, and ultrasound device/model.

Response: We agree that a comprehensive table would enhance transparency.

Action Taken: We have created Table 1, which now includes: study design, sample size, tear type/size, follow-up points, ultrasound technique, parameters assessed, decision type, and level of evidence. In addition, Supplementary Table 1 provides detailed information on ultrasound equipment, transducer frequency, measurement technique, ROI definition, and reliability data for each included study.

Location in Revised Manuscript: Table 1; Supplementary Table 1.

We sincerely thank the editor and all reviewers for their time and constructive comments, which have significantly improved this manuscript. We believe all concerns have been fully addressed and hope the revised version now meets the standards for publication in PLOS ONE.

Yours sincerely,

Chuanxiong Li (Corresponding Author)

Department of Rehabilitation Medicine

The Affiliated Hospital of Yunnan University

176 Qingnian Road, Kunming, Yunnan Province 650021, China

E-mail: 352784777@qq.com
