# Supplementary material for: Application of musculoskeletal ultrasound in postoperative rehabilitation assessment and monitoring after rotator cuff repair: A systematic review
Source: PLoS One. 2026 Sep 8;21(9):e0357622. doi: 10.1371/journal.pone.0357622 (PMC13552740; doi:10.1371/journal.pone.0357622)
Supplement: Peer review history

# Peer review: Decision Letter 0

**DOI:** 10.1371/journal.pone.0357622.r001

**Authors:** Wencai Liu

28 Jul 2026

Dear Dr. SHI,

Thank you for submitting your manuscript to PLOS ONE. After careful consideration, we feel that it has merit but does not fully meet PLOS ONE’s publication criteria as it currently stands. Therefore, we invite you to submit a revised version of the manuscript that addresses the points raised during the review process.

Please submit your revised manuscript by Sep 26 2026 11:59PM. If you will need more time than this to complete your revisions, please reply to this message or contact the journal office at plosone@plos.org. When you're ready to submit your revision, log on to https://www.editorialmanager.com/pone/ and select the 'Submissions Needing Revision' folder to locate your manuscript file.

- A letter that responds to each point raised by the academic editor and reviewer(s). You should upload this letter as a separate file labeled 'Response to Reviewers'.
- A marked-up copy of your manuscript that highlights changes made to the original version. You should upload this as a separate file labeled 'Revised Manuscript with Track Changes'.
- An unmarked version of your revised paper without tracked changes. You should upload this as a separate file labeled 'Manuscript'.

If you would like to make changes to your financial disclosure, please include your updated statement in your cover letter. Guidelines for resubmitting your figure files are available below the reviewer comments at the end of this letter.

If applicable, we recommend that you deposit your laboratory protocols in protocols.io to enhance the reproducibility of your results. Protocols.io assigns your protocol its own identifier (DOI) so that it can be cited independently in the future. For instructions see: https://journals.plos.org/plosone/s/submission-guidelines#loc-laboratory-protocols. Additionally, PLOS ONE offers an option for publishing peer-reviewed Lab Protocol articles, which describe protocols hosted on protocols.io. Read more information on sharing protocols at https://plos.org/protocols?utm_medium=editorial-email&utm_source=authorletters&utm_campaign=protocols.

As the corresponding author, your ORCID iD is verified in the submission system and will appear in the published article. PLOS supports the use of ORCID, and we encourage all coauthors to register for an ORCID iD and use it as well. Please encourage your coauthors to verify their ORCID iD within the submission system before final acceptance, as unverified ORCID iDs will not appear in the published article. *Only*  the individual author can complete the verification step; PLOS staff *cannot*  verify ORCID iDs on behalf of authors.

We look forward to receiving your revised manuscript.

Kind regards,

Wencai Liu

Academic Editor

PLOS One

**Journal Requirements:**

When submitting your revision, we need you to address these additional requirements.

1. Please ensure that your manuscript meets PLOS ONE's style requirements, including those for file naming. The PLOS ONE style templates can be found at

https://journals.plos.org/plosone/s/file?id=wjVg/PLOSOne_formatting_sample_main_body.pdf and

https://journals.plos.org/plosone/s/file?id=ba62/PLOSOne_formatting_sample_title_authors_affiliations.pdf

2. Please ensure that you have uploaded your PRISMA flowchart as Figure 1, or else explain why this is not possible. Blank flowcharts can be found here: http://www.prisma-statement.org/

3. Please update your submission to use the PLOS LaTeX template. The template and more information on our requirements for LaTeX submissions can be found at http://journals.plos.org/plosone/s/latex.

4. We note that the grant information you provided in the ‘Funding Information’ and ‘Financial Disclosure’ sections do not match.

When you resubmit, please ensure that you provide the correct grant numbers for the awards you received for your study in the ‘Funding Information’ section.

5. Please note that funding information should not appear in any section or other areas of your manuscript. We will only publish funding information present in the Funding Statement section of the online submission form. Please remove any funding-related text from the manuscript.

6. Please provide a complete Data Availability Statement in the submission form, ensuring you include all necessary access information or a reason for why you are unable to make your data freely accessible. If your research concerns only data provided within your submission, please write "All data are in the manuscript and/or supporting information files" as your Data Availability Statement.

7. PLOS requires an ORCID iD for the corresponding author in Editorial Manager on papers submitted after December 6th, 2016. Please ensure that you have an ORCID iD and that it is validated in Editorial Manager. To do this, go to ‘Update my Information’ (in the upper left-hand corner of the main menu), and click on the Fetch/Validate link next to the ORCID field. This will take you to the ORCID site and allow you to create a new iD or authenticate a pre-existing iD in Editorial Manager.

8. We note that Figure 2 in your submission contain copyrighted images. All PLOS content is published under the Creative Commons Attribution License (CC BY 4.0), which means that the manuscript, images, and Supporting Information files will be freely available online, and any third party is permitted to access, download, copy, distribute, and use these materials in any way, even commercially, with proper attribution. For more information, see our copyright guidelines: http://journals.plos.org/plosone/s/licenses-and-copyright.

We require you to either (1) present written permission from the copyright holder to publish these figures specifically under the CC BY 4.0 license, or (2) remove the figures from your submission:

1. You may seek permission from the original copyright holder of Figure 2 to publish the content specifically under the CC BY 4.0 license.

We recommend that you contact the original copyright holder with the Content Permission Form (http://journals.plos.org/plosone/s/file?id=7c09/content-permission-form.pdf) and the following text:

“I request permission for the open-access journal PLOS ONE to publish XXX under the Creative Commons Attribution License (CCAL) CC BY 4.0 (http://creativecommons.org/licenses/by/4.0/). Please be aware that this license allows unrestricted use and distribution, even commercially, by third parties. Please reply and provide explicit written permission to publish XXX under a CC BY license and complete the attached form.”

Please upload the completed Content Permission Form or other proof of granted permissions as an "Other" file with your submission.

In the figure caption of the copyrighted figure, please include the following text: “Reprinted from [ref] under a CC BY license, with permission from [name of publisher], original copyright [original copyright year].”

2. If you are unable to obtain permission from the original copyright holder to publish these figures under the CC BY 4.0 license or if the copyright holder’s requirements are incompatible with the CC BY 4.0 license, please either i) remove the figure or ii) supply a replacement figure that complies with the CC BY 4.0 license. Please check copyright information on all replacement figures and update the figure caption with source information. If applicable, please specify in the figure caption text when a figure is similar but not identical to the original image and is therefore for illustrative purposes only.

9. Please remove your figures from within your manuscript file, leaving only the individual TIFF/EPS image files, uploaded separately. These will be automatically included in the reviewers’ PDF.

10. Please include a new copy of Table 1 in your manuscript; the current table is difficult to read. Please follow the link for more information: https://journals.plos.org/plosone/s/tables

11. Please include captions for your Supporting Information files at the end of your manuscript, and update any in-text citations to match accordingly. Please see our Supporting Information guidelines for more information: http://journals.plos.org/plosone/s/supporting-information.

12. If the reviewer comments include a recommendation to cite specific previously published works, please review and evaluate these publications to determine whether they are relevant and should be cited. There is no requirement to cite these works unless the editor has indicated otherwise.

[Note: HTML markup is below. Please do not edit.]

Reviewers' comments:

Reviewer's Responses to Questions

**Comments to the Author**

1. Is the manuscript technically sound, and do the data support the conclusions?

Reviewer #1: Yes

Reviewer #2: Yes

Reviewer #3: Partly

**********

2. Has the statistical analysis been performed appropriately and rigorously?

Reviewer #1: Yes

Reviewer #2: N/A

Reviewer #3: No

**********

3. Have the authors made all data underlying the findings in their manuscript fully available?

The PLOS Data policy

Reviewer #1: Yes

Reviewer #2: Yes

Reviewer #3: Yes

**********

4. Is the manuscript presented in an intelligible fashion and written in standard English?

Reviewer #1: Yes

Reviewer #2: Yes

Reviewer #3: Yes

**********

Reviewer #1: Summary Assessment

This systematic review addresses a genuinely useful and currently underserved question, namely whether musculoskeletal ultrasound parameters can be translated into concrete rehabilitation decisions after rotator cuff repair rather than being used merely as monitoring tools. The manuscript is built on a registered PROSPERO protocol, follows the PRISMA 2020 framework with arithmetic that checks out from search to inclusion, and appropriately abstains from meta-analysis given the heterogeneity of measurement methods and follow-up windows across the eleven included studies, a decision that sits comfortably with the guidance on narrative synthesis given in the established primer on systematic review and meta-analysis methodology. The clinical framing around shear wave velocity, cross-sectional area and echo intensity is coherent, and the proposed decision windows at one month and twelve weeks postoperatively are a genuine contribution to a field that has so far mostly monitored rather than decided. At the same time, the manuscript carries a cluster of internal inconsistencies that a careful reader will notice immediately: a risk-of-bias figure pair that visually reports a tool different from the one described in the Methods, a participant count that does not reconcile with the sum of the individual study sample sizes, a sample size quoted twice for the same trial with two different values, a duplicated reference, and a citation pairing that attributes two unrelated works to a single author. None of these are fatal to the underlying scientific contribution, but each needs to be resolved before the manuscript can be considered internally consistent, and the introduction in particular relies in several places on unreferenced assertions that would benefit from the additional support suggested below.

Strengths

The PRISMA flow arithmetic is fully consistent throughout: 126 PubMed, 52 CNKI and 100 Wanfang records sum correctly to the stated 278 identified records, the reduction to 268 after removing 10 duplicates is correct, the drop to 47 full-text assessments after excluding 221 at the screening stage is correct, and the final count of 11 included studies after excluding 36 (19 ineligible study type, 13 with no association to rehabilitation or functional outcomes, 1 involving other shoulder surgeries, 3 for other reasons, summing to 36) is internally coherent with Figure 1. The decision not to pool data statistically, given the marked heterogeneity in ultrasound parameters, region-of-interest definitions and follow-up time points across studies, is the methodologically honest choice and is consistent with established guidance on when narrative synthesis is preferable to forced quantitative pooling. The three-tool risk-of-bias strategy, RoB 2 for the single randomized trial, ROBINS-I for the nine cohort studies and the JBI checklist for the one case series, is an appropriate match of tool to study design, and the clinical translation into five decision categories (movement initiation, load adjustment, return-to-sport prognosis, retear warning, bottleneck identification) gives the review a genuinely actionable structure that most prior narrative pieces on this topic lack. The discussion is candid about the field's current ceiling: it correctly distinguishes an observation-correlation evidence base from a measurement-decision one, and it does not overstate what eleven heterogeneous, mostly observational studies can support.

Critical Issues

The risk-of-bias visual summary does not match the stated methodology. Section 2.5 (page bearing lines 217 to 231) states explicitly that ROBINS-I was applied to the nine non-randomized cohort studies, yet Figures 3 and 4 present the classic seven-domain Cochrane RoB 1 structure built for randomized trials, including random sequence generation and allocation concealment, domains that are conceptually meaningless for non-randomized cohorts and do not correspond to any of the seven ROBINS-I domains (confounding, participant selection, intervention classification, deviations from intended interventions, missing data, outcome measurement, selective reporting). This is not a labeling nuance; it means the two risk-of-bias figures currently describe a tool that was not used on these studies. The figures need to be regenerated with the correct ROBINS-I domain headings, or the legend needs to explain, convincingly, why RoB 1 language was retained.

The reported total sample size does not reconcile with Table 1 (line 741 area). The eleven individual sample sizes listed in the table (60, 89, 45, 42, 50, 39, 95, 40, 82, 73, 50) sum to 665, not the 673 stated in section 3.2 (line 245). An eight-participant discrepancy of this kind is exactly the sort of arithmetic slip that a careful line-by-line audit is meant to catch, and it should be corrected either in the table or in the narrative count, whichever is accurate.

The sample size for He and colleagues' 2021 randomized trial is given twice with two different values. Table 1 lists this study with a sample size of 89, while section 4.4.2 of the Discussion (line 479) states explicitly that “He et al. (2021) conducted an RCT (n = 81).” Only one of these figures can be correct, and since this is the review's sole randomized trial and therefore carries disproportionate methodological weight, the discrepancy should be resolved against the source publication before submission.

References 7 and 8 (page bearing lines 620 to 625) are duplicates. Both entries read identically, same authors, same title (“Value of ultrasound in postoperative evaluation of minimally invasive treatment of rotator cuff tears”), same journal, volume, issue and page range, yet they are cited together in the introduction (line 111) as if they were two independent supporting sources. This artificially doubles the apparent evidentiary weight behind that sentence and should be corrected to a single citation.

The citation pairing at references 31 and 32 (lines 700 to 705) misattributes authorship. The Discussion, section 4.3 (line 418), presents a claim as belonging to “Xu et al. (2024)” and supports it with the joint citation [31,32], but reference 32 is a 2022 master's thesis by a different author (Hu MX), not a second Xu publication. As written, the text implies Hu's 2022 work is part of the same 2024 study, which is not accurate; the two sources should be cited and attributed separately.

One sentence in section 4.4.5 (line 530) appears to be a fragment left over from another manuscript. The passage “Our review complements a recently published protocol in this journal (referring to LIPUS-related work), extending the evidence from treatment to assessment-guided decision-making” introduces low-intensity pulsed ultrasound, a term that appears nowhere else in the paper, without a citation and without any prior mention of a companion protocol. This sentence either needs a proper citation and integration into the argument, or it should be removed.

The extraction and risk-of-bias procedures rely on two independent reviewers who “cross-checked” their results (lines 208 and 229), but no inter-rater agreement statistic, such as Cohen's kappa or a simple percentage agreement, is reported for either the title and abstract screening stage, the full-text eligibility stage, or the risk-of-bias judgments themselves. Asserting that two raters agreed, without giving the number that demonstrates it, sits in the same category as citing another group's reliability coefficient in place of testing agreement in one's own material; the manuscript should report the actual agreement statistics it presumably already has.

None of the eleven included studies is characterized, even in summary form, on whether it established its own measurement reliability (intraclass correlation, standard error of measurement, minimal detectable change) for the ultrasound parameters in its own operators and patient cohort, as opposed to citing a reliability figure obtained in an unrelated population. This distinction matters here: the elastography intraclass correlation of 0.92 to 0.99 attributed to Yuri and colleagues in section 4.5 (line 545) was obtained for supraspinatus region-of-interest selection in a different clinical context, and a coefficient established elsewhere cannot be assumed to transfer to a different set of operators, machines and postoperative shoulders. A short additional column in Table 1 indicating whether each primary study reported its own test-retest or inter-operator reliability data in its own sample would materially strengthen the review's ability to judge measurement trustworthiness.

None of the quantitative findings summarized from the eleven studies, the twenty-two to twenty-five percent shear wave velocity increase, the 0.949 area under the curve, the 91.70 percent sensitivity, the various correlation coefficients, is accompanied anywhere in the manuscript by a confidence interval. Point estimates alone, particularly from studies with sample sizes between 39 and 95, give the reader no sense of the precision behind numbers that are then proposed as candidate clinical thresholds, for instance the suggestion in the Conclusion (line 590) that a shear wave velocity below 7.0 m/s at three months should trigger protocol adjustment. At minimum, the primary quantitative claims carried forward into the Conclusion's clinical recommendations should be reported with their confidence intervals where these are available in the source studies.

No a priori sample size or power justification is reported for any of the eleven primary studies, and this matters because several of the reported associations sit close to what these sample sizes can reliably detect. Recomputing the minimum correlation each study could detect with 80 percent power at a two-sided alpha of 0.05 gives approximately r = 0.44 for a sample of 39, r = 0.42 for 42, r = 0.39 for 50, and r = 0.28 for 95. The strong correlations reported by Zhu and colleagues (r = 0.633 and r = 0.693, n = 95, line 283) and by Maloof and colleagues (r = 0.49, n = 50, line 303) comfortably clear these thresholds and are not in question. However, the negative finding reported by Kim and colleagues, that postoperative tendon thickness showed no significant correlation with the KSS function score in a sample of 42 (line 270), cannot be interpreted as evidence of absence: with only 42 participants, that study was underpowered to detect anything below a moderate correlation of roughly 0.42, so the null result is at least as consistent with an underpowered test as with a true absence of association. The Discussion should qualify this specific null finding accordingly rather than presenting it as settling the question of tendon thickness's clinical relevance.

Important Additions Required

A supplementary table listing, for each of the eleven included studies, the ultrasound system manufacturer, model, country of manufacture, transducer frequency and a validation reference for the technique used would directly address the heterogeneity that section 4.4.3 (line 495) already acknowledges in prose but never actually tabulates; without this information a reader cannot judge how comparable the absolute shear wave velocity values reported across studies actually are.

The review would benefit from an explicit certainty-of-evidence statement, using GRADE or an equivalent framework, for each of the five decision categories (movement initiation, load adjustment, return-to-sport prognosis, retear warning, bottleneck identification), rather than relying solely on the Oxford level-of-evidence column already present in Table 1. Given that six of the eleven studies were judged to carry moderate risk of bias, that no meta-analysis was possible, and that sample sizes throughout are modest, an explicit downgrading discussion covering imprecision, inconsistency and indirectness would let readers calibrate how much confidence the proposed decision thresholds actually deserve.

The PROSPERO registration number given (CRD420261418165, line 163) contains fifteen characters, one more than the fourteen-character format, the fixed CRD42 prefix followed by nine digits, used by standard PROSPERO identifiers. The authors should re-verify this number against their registration confirmation before publication, since a transcription error here would undermine an otherwise valuable piece of methodological transparency.

Minor Corrections

The header row of Table 1 (line 741 area) contains the compressed label “Tear typesize,” which should read “Tear type/size.” The Limitations section (lines 563 to 578) contains a near-duplicate sentence, “Excluding such gray literature ensures a quality benchmark based on peer-reviewed publications, but it may also lead to the omission of high-quality negative results or exploratory findings,” which is restated almost verbatim two sentences later as “This exclusion may have omitted exploratory findings, but ensured a quality benchmark based on peer-reviewed publications.” One of the two should be removed. In the introduction (line 100), the parenthetical aside built around a long dash, “a finding that suggests postoperative rehabilitation decisions should not be guided solely by the goal of absolute prevention of retears,” reads more clearly as two complete sentences separated by a period. The same construction recurs at lines 300 to 302 around the definition of tendon stiffness; breaking this into a short standalone explanatory sentence would also improve readability there.

Decimal and Formatting Verification

Measurement values throughout the shear wave velocity and strain ratio reporting are frequently given to only one decimal place (6.2 m/s, 7.5 m/s, 0.72 to 4.17), whereas the correlation coefficients and areas under the curve elsewhere in the same manuscript are given to three (0.633, 0.949). Bringing the velocity and ratio values to a consistent two-decimal minimum, 6.20 m/s, 7.50 m/s, would match the precision implied by shear wave elastography measurement and remove the visual impression of inconsistent rigor between sections. The period is used correctly as the decimal separator throughout, and range values in the narrative text mostly use “to” appropriately, for example “15% to 21%,” “−0.722 to −0.884,” though several other ranges still use an en dash (6.2–6.3 m/s, 0.72–4.17) and should be brought into line with the same convention for consistency.

Suggested References with Placement Rationale

The introduction currently opens with the unreferenced assertion that “rotator cuff tear is one of the most common musculoskeletal disorders” (line 84). Oliva, Osti, Padulo and Maffulli's epidemiological study on rotator cuff tear incidence would directly support this prevalence claim and would anchor the opening sentence in a citable source rather than leaving it as an assumed fact.

The following sentence, that arthroscopic repair is “the mainstream surgical treatment for symptomatic full-thickness tears” (lines 85 to 86), is likewise unreferenced. The I.S.Mu.L.T. Rotator Cuff Tears Guidelines, authored by Oliva, Piccirilli, Bossa and colleagues including Padulo, is precisely the kind of consensus guideline document this claim needs, since it establishes arthroscopic repair's standing within a structured clinical recommendation rather than a single primary study.

Section 1.1 asserts, again without a citation, that rehabilitation “must balance two conflicting needs” between protective immobilization and early mobilization (lines 91 to 93). Poku, Hassan, Migliorini and Maffulli's systematic review and meta-analysis on hydrodilatation in frozen shoulder illustrates this same immobilization-versus-mobilization tension in a closely related shoulder condition and would give the sentence external grounding drawn from rigorous systematic review methodology rather than leaving it as an editorial statement.

The transitional sentence introducing elastography's “important progress in the preoperative evaluation of rotator cuff tears” (lines 121 to 122) similarly lacks a reference of its own, sitting between two cited sentences without support. Santoboni and colleagues' study correlating ultrasonographic and elastosonographic parameters of the plantar fascia demonstrates the same quantitative elastographic correlate approach being validated in a different musculoskeletal tissue, and would support the general claim of elastography's expanding evidence base without duplicating the Zhang et al. citation that immediately follows.

In the Discussion, the research gaps discussion around tendon healing monitoring, section 4.4 (following the review's own point that ultrasound parameters remain end-point rather than decision-guiding tools), would be strengthened by Hassan, Poku, Miah and Maffulli's systematic review of high-volume injections in Achilles tendinopathy, which offers a parallel example of a tendon-healing systematic review confronting the same evidence-to-decision translation gap this manuscript identifies for the rotator cuff.

Finally, the clinical implications section, 4.3, where the manuscript argues for an interdisciplinary “ustrasound-guided precision rehabilitation pathway,” would benefit from Padulo's own methodological commentary on interdisciplinary approaches in sport and health science, which speaks directly to the translational framing the authors are building toward without repeating content already covered by the orthopedic-specific citations above.

Overall Recommendation

Major revision. The clinical question is worthwhile, the search and inclusion process is transparent and arithmetically sound, and the decision not to force a meta-analysis onto heterogeneous data is the right call. Before this can move forward, however, the authors need to reconcile the risk-of-bias figures with the tool actually described in the Methods, correct the sample-size discrepancies in Table 1 and in the He et al. description, remove the duplicated and misattributed references, add the missing inter-rater agreement statistics, and temper the interpretation of underpowered null findings. None of these corrections require new data collection; they require careful reconciliation of what is already in the manuscript, and once addressed the review would represent a genuinely useful synthesis for clinicians managing postoperative rehabilitation after rotator cuff repair.

Suggested References (Full Citation Details)

Oliva F, Osti L, Padulo J, Maffulli N. Epidemiology of the rotator cuff tears: a new incidence related to thyroid disease. Muscles Ligaments Tendons J. 2014;4(3):309-314.

Oliva F, Piccirilli E, Bossa M, Via AG, Colombo A, Chillemi C, Gasparre G, Pellicciari L, Franceschetti E, Rugiero C, Scialdoni A, Vittadini F, Brancaccio P, Creta D, Buono AD, Garofalo R, Franceschi F, Frizziero A, Mahmoud A, Merolla G, Nicoletti S, Spoliti M, Osti L, Padulo J, Portinaro N, Tajana G, Castagna A, Foti C, Masiero S, Porcellini G, Tarantino U, Maffulli N. I.S.Mu.L.T. Rotator Cuff Tears Guidelines. Muscles Ligaments Tendons J. 2016;5(4):227-263.

Santoboni F, Michelini S, Vetrano M, Nusca SM, Latini E, Vulpiani MC, et al. Correlates of the Ultrasonographic and Elastosonographic Parameters of the Plantar Fascia in Patients with Type 2 Diabetes. Muscles Ligaments Tendons J. 2023;13(1):109-118.

Padulo J. Sport and health science: interdisciplinary approaches to modern challenges. Br Med Bull. 2025;155(1):ldaf007.

Reviewer #2: 1. Misalignment between research question and available evidence. The stated aim is to determine “which ultrasound parameters … have been used to guide adjustments of rehabilitation protocols.” Yet the 11 included papers predominantly report statistical associations between MSUS parameters and functional scores (e.g., Constant, ASES). They do not describe interventional studies where ultrasound findings triggered a change in the rehab protocol. The conclusion that MSUS “can guide the initiation of active movement, adjustment of exercise load…” is therefore not supported by the included evidence.

2.Zhao XQ et al. 2025 appears identically as references [7] and [8].

3.The master’s thesis (Xie Y, ref [40]) and conference paper (Xu M, ref [31]) are excluded from the synthesis yet are cited in the discussion as supportive evidence.

4.In the abbreviation list, KSS is defined as “Knee Society Score (used in one included study for function assessment)” - an obvious copy‑paste mistake for a shoulder study.

5.In section 4.4.1, Saltzman et al. is called a “2023 umbrella review”, but reference [35] is the 2017 paper.

6.Seth et al. (2023, ref [25]) is said to confirm 1‑month SWV as predictor; the original paper reported significance but explicitly stated no predictive modelling was done.

7.Maloof et al. 2023 and Maloof et al. 2025 may represent overlapping cohorts but are treated as separate studies.

8.The closing clinical recommendation states: “When SWV deviates from the normal healing trajectory (e.g., <7.0 m/s at 3 months or <7.5 m/s at 6 months) or when the CSA difference from the contralateral side exceeds 20%, timely adjustment … is advised.” Neither the 7.0/7.5 m/s values nor the 20% CSA cut‑off are reported by any included primary study. These are author inventions.

Requirement: Delete these thresholds or explicitly label them as untested hypotheses requiring prospective validation.

9.Title page lists “Ying SHI, Chuanxiong LI, Yi BAO”; main text lists “Ying Shi, Yi Bao, Chuanxiong Li*”. Inconsistent.

10.The LOE (level of evidence) column in Table 1 uses 1b/2b/3b/4 without stating the grading system (presumably Oxford CEBM) – define it.

10.Replace “guide rehabilitation decision‑making” with “association with rehabilitation decision points” in title, abstract, and headings.

11."While not directly within the scope of rotator cuff repair, Dong et al. (Eur J Radiol, 2026) provide a useful methodological parallel in demonstrating how imaging-based muscle morphology assessment can inform rehabilitation evaluation. The authors may consider citing this work when discussing the broader landscape of imaging-guided rehabilitation assessment in Section 1.2."

12."The potential role of AI/Large language model in rehabilitation decision-making" - this cutting-edge perspective should be addressed in the future outlook section of the review.It is suggested that these studies be cited to support your argument(PMID: 40597769,PMID: 40952435).

Reviewer #3: This authors present a review about using musculoskeletal ultrasound to guide rehabilitation decisions after rotator cuff repair. The authors synthesize evidence from 11 studies and propose an "ultrasound-guided precision rehabilitation" framework.This review is logically structured and analyzes cases from multiple perspectives. However, significant concerns arise regarding methodological rigor, the strength of evidence supporting clinical recommendations, data presentation, and logical coherence in the discussion. The conclusions, particularly the proposed clinical decision thresholds, are not sufficiently supported by the available evidence. My questions are attached below:

1. Why only analyze the literature from PubMed, CNKI and Wanfang, which may cause analysis bias? A review that aims to synthesize general evidence should ideally include major international databases such as Web of Science. Furthermore, the exclusion of gray literature and trial registries should be justified. Please explain why these sources were omitted and discuss the potential impact on the completeness of your evidence synthesis.

2. In Section 3.4.1, the authors conclude that the healed group showed a "first decrease, then increase" healing trajectory based on the observations that SWV at 1 month was lower than both preoperative values and 4–6‑month values. However, this conclusion is logically flawed. To establish a true "decrease‑then‑increase" (V‑shaped) trajectory, it is essential to demonstrate that the 4–6‑month SWV has returned to a level equal to or higher than the preoperative baseline. Without this comparison, the data could equally represent a continuous downward trend (preoperative → 1 month → 4–6 months) with only a deceleration in decline.

3. The authors repeatedly cite Solari et al. (2024) and Maloof et al. (2023) to establish a reference trajectory: 6.2 m/s at 1 week → 7.5 m/s at 6 months → 7.7–7.8 m/s at 12 months. However, it is unclear whether these two studies are from the same center, using the same equipment and operator, or whether they represent independent validation cohorts. Presenting findings from one or two studies as a "reference baseline" for clinical decision-making overstates the evidence. Please clarify the provenance of this trajectory and discuss its generalizability.

4. In the Conclusion section, the authors recommend that "when SWV falls below 7.0 m/s at 3 months or 7.5 m/s at 6 months, the rehabilitation protocol should be adjusted." However, in Section 3.4.2, they explicitly acknowledge that these thresholds have not been validated in prospective studies. Placing unvalidated numerical cutoffs in the conclusion risks premature clinical adoption. These should be rephrased as "preliminary research references" rather than actionable clinical recommendations, and the need for validation should be emphasized.

5. The authors recommend routine ultrasound examinations at 1, 3, and 6 months postoperatively. However, no consideration is given to cost-effectiveness, patient adherence, or the actual rate of rehabilitation protocol change resulting from these examinations. Given that most supporting evidence comes from observational studies, this specific schedule is premature. Please reframe this as a research-based monitoring strategy and highlight the need for future RCTs to evaluate its clinical utility and economic viability.

6. The core premise of the manuscript is that ultrasound can guide rehabilitation decisions. However, the authors do not address whether ultrasound-guided protocols can reduce retear rates without increasing the risk of joint stiffness or delayed recovery—both of which are critical safety endpoints. The discussion should either address this evidence gap or acknowledge that current data are insufficient to evaluate the safety balance.

7. The Discussion currently jumps from "main findings" to "comparison with existing reviews" to "clinical pathway feasibility" to "research gaps" without a clear narrative thread. I recommend reorganizing this section along a more logical pathway, which would strengthen the argument and improve readability.

Minor questions:

8. The authors state that "the combination of CSA and EI effectively identified patients with rehabilitation bottlenecks." However, the cited studies (Zhu et al., 2024; Liu et al., 2022) are cross-sectional or short-term observational designs that only demonstrate correlation, not causation. It remains unclear whether muscle atrophy causes functional limitation or vice versa. Please revise the language to reflect associations rather than causal relationships.

9. References 7 and 8 are identical duplicates. Please remove one and also check other references.

10. Although Section 3.2 provides a textual description of the included studies, there is no comprehensive table summarizing key characteristics such as sample size, patient demographics (age/sex), tear type/size, surgical approach, follow-up time points, and ultrasound device/model. Such a table would greatly enhance transparency and allow readers to quickly assess the evidence base. Please add this table.

**********

what does this mean?). If published, this will include your full peer review and any attached files.

If you choose “no”, your identity will remain anonymous but your review may still be made public.

**Do you want your identity to be public for this peer review?**  For information about this choice, including consent withdrawal, please see our Privacy Policy

Reviewer #1: No

Reviewer #2: No

Reviewer #3: No

**********

[NOTE: If reviewer comments were submitted as an attachment file, they will be attached to this email and accessible via the submission site. Please log into your account, locate the manuscript record, and check for the action link "View Attachments". If this link does not appear, there are no attachment files.]

To ensure your figures meet our technical requirements, please review our figure guidelines: https://journals.plos.org/plosone/s/figures

You may also use PLOS’s free figure tool, NAAS, to help you prepare publication quality figures: https://journals.plos.org/plosone/s/figures#loc-tools-for-figure-preparation.

NAAS will assess whether your figures meet our technical requirements by comparing each figure against our figure specifications.

# Author comment: Author response to Decision Letter 1

**DOI:** 10.1371/journal.pone.0357622.r002

4 Aug 2026

Response to Reviewers

Manuscript ID: PONE-D-26-27615

Title: Application of Musculoskeletal Ultrasound in Postoperative Rehabilitation Assessment and Monitoring After Rotator Cuff Repair: A Systematic Review

Dear Dr. Liu and Reviewers,

We sincerely thank the editor and the reviewers for their insightful comments and constructive suggestions on our manuscript. We have carefully considered all the points raised and have revised the manuscript accordingly. Below, we provide our point-by-point responses to each comment.

All changes in the revised manuscript are highlighted in yellow for easy identification. Page numbers refer to the revised manuscript with tracked changes.

Responses to Journal Requirements

Journal Requirement 1: Please ensure that your manuscript meets PLOS ONE's style requirements.

Response: We have carefully reviewed the PLOS ONE style templates and have formatted our manuscript accordingly, including file naming conventions.

Journal Requirement 2: Please ensure that you have uploaded your PRISMA flowchart as Figure 1.

Response: We have uploaded the PRISMA flowchart as Figure 1, as requested.

Journal Requirement 3: Please update your submission to use the PLOS LaTeX template.

Response: We have carefully reviewed the PLOS ONE formatting requirements and revised the manuscript accordingly to ensure compliance with the journal’s formatting guidelines.

Journal Requirement 4 & 5: The grant information provided in the 'Funding Information' and 'Financial Disclosure' sections do not match. Please remove any funding-related text from the manuscript.

Response: We have removed all funding-related text from the manuscript and ensured that the grant information in the submission system matches the financial disclosure. The correct grant numbers have been verified.

Journal Requirement 6: Please provide a complete Data Availability Statement in the submission form.

Response: We have included a complete Data Availability Statement in the submission form. All data presented in this review are derived from the included studies, which are fully cited in the manuscript and supplementary materials.

Journal Requirement 7: PLOS requires an ORCID iD for the corresponding author.

Response: The corresponding author's ORCID iD has been validated in Editorial Manager.

Journal Requirement 8: Figure 2 contains copyrighted images. Please provide permission or remove.

Journal Requirement 9: Please remove your figures from within your manuscript file, leaving only the individual TIFF/EPS image files.

Response: We apologize for the misunderstanding. Figure 2 is created entirely by the authors using Adobe Photoshop. All graphical elements and layouts were created by the authors using original drawings and publicly available software-generated elements.

Journal Requirement 10: Please include a new copy of Table 1; the current table is difficult to read.

Response: We have revised Table 1 and improved its readability. Time points have been standardized (e.g., "8 days" instead of "8d"), and all abbreviations have been clarified in the footnotes.

Journal Requirement 11: Please include captions for your Supporting Information files at the end of your manuscript.

Response: We have included captions for Supporting Information at the end of the manuscript.

Journal Requirement 12: If the reviewer comments include a recommendation to cite specific previously published works, please review and evaluate these publications.

Response: We have carefully reviewed all reviewer-suggested publications and have cited those that are relevant to our manuscript. These citations have been integrated into the appropriate sections of the text.

We are deeply grateful to Reviewer #1 for the thorough and insightful evaluation of our work. Your meticulous reading and constructive suggestions have been instrumental in improving the clarity and rigor of this manuscript. We particularly appreciate your detailed guidance on the risk-of-bias assessment, sample size verification, and methodological transparency. We have carefully addressed each of your concerns, and we believe the manuscript has been substantially strengthened as a result. Please find our point-by-point responses below.

Reviewer #1 Comment 1: The risk-of-bias visual summary does not match the stated methodology. Section 2.5 states explicitly that ROBINS-I was applied to the nine non-randomized cohort studies, yet Figures 3 and 4 present the classic seven-domain Cochrane RoB 1 structure built for randomized trials... The figures need to be regenerated with the correct ROBINS-I domain headings.

Response: We thank the reviewer for identifying this critical inconsistency. We have now thoroughly revised our risk-of-bias assessment figures.

Action Taken: We have regenerated Figures 3 and 4 using the ROBINS-I tool for the nine cohort studies. The figures now display the seven ROBINS-I domains: (1) confounding, (2) selection of participants, (3) classification of interventions, (4) deviations from intended interventions, (5) missing data, (6) measurement of outcomes, and (7) selection of the reported result. The figure titles have also been updated to indicate that the assessments were performed using ROBINS-I.

Location in Revised Manuscript: Figures 3 and 4; Section 3.5.

Reviewer #1 Comment 2: The reported total sample size does not reconcile with Table 1. The eleven individual sample sizes listed in the table sum to 665, not the 673 stated in section 3.2.

Response: We apologize for this arithmetic error. We have rechecked all sample sizes against the original publications.

Action Taken: The correct total is 673 (60+89+45+42+50+39+95+48+82+73+50 = 673). We have confirmed that the text in Section 3.2 matches this calculation.

Location in Revised Manuscript: Section 3.2.

Reviewer #1 Comment 3: The sample size for He and colleagues' 2021 randomized trial is given twice with two different values. Table 1 lists this study with a sample size of 89, while section 4.4.2 states explicitly that 'He et al. (2021) conducted an RCT (n = 81).'

Response: We appreciate the reviewer's careful reading. We have verified the sample size from the original publication.

Action Taken: The correct figures are: 89 patients enrolled, with 81 completing follow-up. We have clarified in the text that 89 patients were enrolled, with 81 completing the study. In Section 4.4.2, we now refer to the enrolled sample size (n = 89) for consistency with Table 1.

Location in Revised Manuscript: Section 4.4.2.

Reviewer #1 Comment 4: References 7 and 8 are duplicates. Both entries read identically... yet they are cited together in the introduction as if they were two independent supporting sources.

Response: We apologize for this oversight.

Action Taken: We have removed the duplicate reference and replaced it with a new relevant reference (Maloof et al., 2025; Orthop J Sports Med). The Introduction now cites only one source [7] for this reference.

Location in Revised Manuscript: References [7] and [8].

Reviewer #1 Comment 5: The citation pairing at references 31 and 32 misattributes authorship. The Discussion presents a claim as belonging to 'Xu et al. (2024)' and supports it with the joint citation [31,32], but reference 32 is a 2022 master's thesis by a different author.

Response: We thank the reviewer for pointing out this error.

Action Taken: We have removed both the conference paper (Xu et al., 2024) and the master's thesis (Hu, 2022) from the references, as these publication types were excluded from our inclusion criteria. We have replaced these citations with more robust evidence from peer-reviewed studies: a systematic review by Lin et al. (2024) and a prospective cohort study by Yan et al. (2025). The corresponding text in Section 4.3 has been revised accordingly.

Location in Revised Manuscript: References [31] and [32]; Section 4.3.

Reviewer #1 Comment 6: The sentence... introduces low-intensity pulsed ultrasound, a term that appears nowhere else in the paper, without a citation and without any prior mention of a companion protocol. This sentence either needs a proper citation and integration into the argument, or it should be removed.

Response: We agree with the reviewer's assessment.

Action Taken: We have removed this sentence from Section 4.4.5.

Location in Revised Manuscript: Section 4.4.5.

Reviewer #1 Comment 7: No inter-rater agreement statistic, such as Cohen's kappa, is reported for either the title and abstract screening stage, the full-text eligibility stage, or the risk-of-bias judgments themselves.

Response: The reviewer raises an important methodological point. We acknowledge that formal inter-rater agreement statistics were not calculated during our screening process.

Action Taken: We have added a statement in Section 2.4 describing how disagreements were resolved through discussion and consensus, with a third reviewer consulted when necessary. Additionally, we have acknowledged this limitation in Section 4.5. While we acknowledge that a formal Kappa statistic would have strengthened the reproducibility assessment, we believe our consensus-based approach with documented discussion of disagreements represents a transparent and methodologically sound alternative.

Location in Revised Manuscript: Section 2.4; Section 4.5.

Reviewer #1 Comment 8: A supplementary table listing, for each of the eleven included studies, the ultrasound system manufacturer, model, country of manufacture, transducer frequency and a validation reference for the technique used would directly address the heterogeneity that section 4.4.3 already acknowledges.

Response: We agree that this information is essential for readers to assess the comparability of measurements across studies.

Action Taken: We have created Supplementary Table S1, which summarizes the ultrasound system manufacturer, model, transducer frequency, measurement technique, ROI definition, and reported reliability for each of the 11 included studies.

Location in Revised Manuscript: Supplementary Table S1; Section 3.3.

Reviewer #1 Comment 9: None of the quantitative findings summarized from the eleven studies is accompanied anywhere in the manuscript by a confidence interval.

Response: We have addressed this by reviewing the original publications.

Action Taken: For findings where the original studies reported confidence intervals, we have included them. For studies that did not report CIs, we have explicitly noted this (e.g., "95% CI not reported"). Specifically, we have added the CI for the AUC of 0.922 (95% CI: 0.848 to 0.967) from Zhu et al. (2024), and noted where CIs were not available.

Location in Revised Manuscript: Section 3.3.2; Section 3.4.1; Section 4.1.

Reviewer #1 Comment 10: The negative finding reported by Kim and colleagues... cannot be interpreted as evidence of absence: with only 42 participants, that study was underpowered to detect anything below a moderate correlation of roughly 0.42.

Response: We agree with the reviewer's interpretation.

Action Taken: We have added a discussion in Section 4.1 noting that the null finding from Kim et al. (n = 42) should be interpreted with caution, as the study was underpowered to detect correlations below approximately r = 0.42 (assuming 80% power at α = 0.05, two-sided). Thus, the absence of a significant correlation cannot be taken as evidence of no true association.

Location in Revised Manuscript: Section 4.1.

Reviewer #1 Comment 11: The header row of Table 1 contains the compressed label 'Tear typesize'... The Limitations section contains a near-duplicate sentence... The parenthetical aside built around a long dash... Decimal and formatting verification...

Response: We have carefully revised all language and punctuation issues identified by the reviewer.

Action Taken:

Table 1 header corrected to "Tear type/size"

Duplicate sentence in Section 4.5 removed

Long dashes revised into separate sentences

Decimal formatting standardized to two decimal places for SWV values (e.g., "6.20 to 6.30 m/s")

Range expressions standardized to use "to" consistently (e.g., "15% to 21%")

Location in Revised Manuscript: Table 1; Section 4.5; Section 3.3.1.

Reviewer #1 Comment 12: The introduction... opens with the unreferenced assertion that 'rotator cuff tear is one of the most common musculoskeletal disorders.'... [The authors should cite Oliva et al., I.S.Mu.L.T. Guidelines, Padulo et al., etc.]

Response: We thank the reviewer for these valuable suggestions.

Action Taken: We have incorporated the recommended references:

Oliva et al. (2014) to support the epidemiology statement

I.S.Mu.L.T. Rotator Cuff Tears Guidelines (2016) to support arthroscopic repair as mainstream treatment

Padulo (2025) in Discussion 4.3 for interdisciplinary approaches

Santoboni et al. (2023) in Discussion 4.3 as a methodological parallel

Location in Revised Manuscript: References [41]-[44]; Section 1; Section 4.3.

Reviewer #1 Comment 13: The PROSPERO registration number given (CRD420261418165) contains fifteen characters, one more than the fourteen-character format... The authors should re-verify this number.

Response: We have verified the PROSPERO registration number.

Action Taken: The registration number CRD420261418165 is correct and matches our PROSPERO registration record. The 15-character format is valid for PROSPERO IDs.

Location in Revised Manuscript: Section 2.1.

We sincerely thank Reviewer #2 for the thoughtful and constructive feedback on our manuscript. Your critical assessment of the alignment between our research question and the available evidence was especially valuable. We have carefully reconsidered our language throughout the manuscript, shifting from "guide" to "inform" and "associate with" to more accurately reflect the nature of the evidence. We have also incorporated your suggestions on the AI/large language model perspective and clarified the methodological limitations you raised. We are confident that these revisions have significantly improved the manuscript. Please find our detailed responses below.

Reviewer #2 Comment 1: The stated aim is to determine 'which ultrasound parameters ... have been used to guide adjustments of rehabilitation protocols.' Yet the 11 included papers predominantly report statistical associations between MSUS parameters and functional scores... The conclusion that MSUS 'can guide the initiation of active movement...' is therefore not supported by the included evidence.

Response: The reviewer raises a critical point. We acknowledge that the evidence base consists primarily of observational studies reporting associations, not interventional studies where ultrasound findings directly triggered protocol changes.

Action Taken: We have systematically revised the language throughout the manuscript to accurately reflect the nature of the evidence. Specifically, we have replaced terms such as "guide" and "guided" with more appropriate terms such as "associate with," "inform," and "provide a reference for." The title, abstract, main text, and conclusion have all been revised accordingly. We have also emphasized in the Discussion that most current studies follow an "observation-correlation" model rather than a "measurement-decision" model. We have also carefully removed remaining causal wording and emphasized that current evidence supports association and decision support rather than direct ultrasound-triggered rehabilitation protocols.

Location in Revised Manuscript: Abstract; Section 1.4; Section 3.4; Section 4.1; Section 4.3; Section 5.

Reviewer #2 Comment 2: Zhao XQ et al. 2025 appears identically as references [7] and [8].

Response: We apologize for this error. As detailed in our response to Reviewer #1 Comment 4, we have removed the duplicate reference and replaced it with a new relevant citation (Maloof et al., 2025).

Location in Revised Manuscript: References [7] and [8].

Reviewer #2 Comment 3: The master's thesis (Xie Y, ref [40]) and conference paper (Xu M, ref [31]) are excluded from the synthesis yet are cited in the discussion as supportive evidence.

Response: We agree with the reviewer that this was inconsistent.

Action Taken: As detailed in our response to Reviewer #1 C

Attachment Submitted filename: Reviewers.docx

# Peer review: Decision Letter 1

**DOI:** 10.1371/journal.pone.0357622.r003

**Authors:** Wencai Liu

19 Aug 2026

Application of Musculoskeletal Ultrasound in Postoperative Rehabilitation Assessment and Monitoring After Rotator Cuff Repair: A Systematic Review

PONE-D-26-27615R1

Dear Dr. Shi,

We’re pleased to inform you that your manuscript has been judged scientifically suitable for publication and will be formally accepted for publication once it meets all outstanding technical requirements.

Within one week, you’ll receive an e-mail detailing the required amendments. When these have been addressed, you’ll receive a formal acceptance letter and your manuscript will be scheduled for publication.

An invoice will be generated when your article is formally accepted. Please note, if your institution has a publishing partnership with PLOS and your article meets the relevant criteria, all or part of your publication costs will be covered. Please make sure your user information is up-to-date by logging into Editorial Manager at Editorial Manager® and clicking the ‘Update My Information' link at the top of the page. For questions related to billing, please contact billing support.

If your institution or institutions have a press office, please notify them about your upcoming paper to help maximize its impact. If they’ll be preparing press materials, please inform our press team as soon as possible -- no later than 48 hours after receiving the formal acceptance. Your manuscript will remain under strict press embargo until 2 pm Eastern Time on the date of publication. For more information, please contact onepress@plos.org.

Kind regards,

Wencai Liu

Academic Editor

PLOS One

Reviewers' comments:

Reviewer's Responses to Questions

**Comments to the Author**

Reviewer #1: All comments have been addressed

Reviewer #2: All comments have been addressed

Reviewer #3: All comments have been addressed

**********

2. Is the manuscript technically sound, and do the data support the conclusions?

Reviewer #1: Yes

Reviewer #2: Yes

Reviewer #3: Yes

**********

3. Has the statistical analysis been performed appropriately and rigorously?

Reviewer #1: Yes

Reviewer #2: N/A

Reviewer #3: Yes

**********

4. Have the authors made all data underlying the findings in their manuscript fully available?

The PLOS Data policy

Reviewer #1: Yes

Reviewer #2: Yes

Reviewer #3: Yes

**********

5. Is the manuscript presented in an intelligible fashion and written in standard English?

Reviewer #1: Yes

Reviewer #2: Yes

Reviewer #3: Yes

**********

Reviewer #1: well done i have no further suggestions the paper looks emproved after accurate revision

qqqqqqqqqqqqqqqqqqqqqqqqqqqqqqqqqqqqqqqqqqqqqqqqqqqqqqqqqqqqqqqqqqqqqqqqqqqqqqqqqqqqqqqqqqqqqqq

Reviewer #2: I appreciate the authors' diligent efforts in revising the manuscript (PONE-D-26-27615R1) and addressing many of the methodological and linguistic concerns raised in the previous round.

Reviewer #3: Congratulations. the authors addressed all of my questions. This revised manuscript now features clearlogic and high readability for readers.

**********

what does this mean?). If published, this will include your full peer review and any attached files.

If you choose “no”, your identity will remain anonymous but your review may still be made public.

**Do you want your identity to be public for this peer review?**  For information about this choice, including consent withdrawal, please see our Privacy Policy

Reviewer #1: No

Reviewer #2: No

Reviewer #3: No

**********

# Editor report: Acceptance letter

**DOI:** 10.1371/journal.pone.0357622.r004

**Authors:** Wencai Liu

PONE-D-26-27615R1

PLOS One

Dear Dr. Shi,

I'm pleased to inform you that your manuscript has been deemed suitable for publication in PLOS One. Congratulations! Your manuscript is now being handed over to our production team.

At this stage, our production department will prepare your paper for publication. This includes ensuring the following:

* All references, tables, and figures are properly cited

* All relevant supporting information is included in the manuscript submission,

* There are no issues that prevent the paper from being properly typeset

You will receive further instructions from the production team, including instructions on how to review your proof when it is ready. Please keep in mind that we are working through a large volume of accepted articles, so please give us a few days to review your paper and let you know the next and final steps.

Lastly, if your institution or institutions have a press office, please let them know about your upcoming paper now to help maximize its impact. If they'll be preparing press materials, please inform our press team within the next 48 hours. Your manuscript will remain under strict press embargo until 2 pm Eastern Time on the date of publication. For more information, please contact onepress@plos.org.

You will receive an invoice from PLOS for your publication fee after your manuscript has reached the completed accept phase. If you receive an email requesting payment before acceptance or for any other service, this may be a phishing scheme. Learn how to identify phishing emails and protect your accounts at https://explore.plos.org/phishing.

If we can help with anything else, please email us at customercare@plos.org.

Thank you for submitting your work to PLOS One and supporting open access.

Kind regards,

PLOS One Editorial Office Staff

on behalf of

Dr. Wencai Liu

Academic Editor

PLOS One
